# A Comprehensive Resource of Interacting Protein Regions for Refining Human Transcription Factor Networks

**DOI:** 10.1371/journal.pone.0009289

**Published:** 2010-02-24

**Authors:** Etsuko Miyamoto-Sato, Shigeo Fujimori, Masamichi Ishizaka, Naoya Hirai, Kazuyo Masuoka, Rintaro Saito, Yosuke Ozawa, Katsuya Hino, Takanori Washio, Masaru Tomita, Tatsuhiro Yamashita, Tomohiro Oshikubo, Hidetoshi Akasaka, Jun Sugiyama, Yasuo Matsumoto, Hiroshi Yanagawa

**Affiliations:** 1 Advanced Research Centers, Keio University, Yokohama, Japan; 2 Department of Biosciences and Informatics, Faculty of Science and Technology, Keio University, Yokohama, Japan; 3 Department of Environment and Information Studies, Keio University, Fujisawa, Japan; 4 Systems Biology Program, Graduate School of Media and Governance, Keio University, Fujisawa, Japan; 5 BioIT Business Development Unit, Fujitsu Limited, Chiba, Japan; 6 Production Solution Business Unit, Production Solution Division, Solutions and Services Department, Fujitsu Advanced Engineering Limited, Tokyo, Japan; 7 Special Suite Team, Custom Primer Production Department, Haneda Laboratories, Invitrogen Japan K.K., Tokyo, Japan; 8 Automation, QIAGEN K.K., Tokyo, Japan; Virginia Tech, United States of America

## Abstract

Large-scale data sets of protein-protein interactions (PPIs) are a valuable resource for mapping and analysis of the topological and dynamic features of interactome networks. The currently available large-scale PPI data sets only contain information on interaction partners. The data presented in this study also include the sequences involved in the interactions (i.e., the interacting regions, IRs) suggested to correspond to functional and structural domains. Here we present the first large-scale IR data set obtained using mRNA display for 50 human transcription factors (TFs), including 12 transcription-related proteins. The core data set (966 IRs; 943 PPIs) displays a verification rate of 70%. Analysis of the IR data set revealed the existence of IRs that interact with multiple partners. Furthermore, these IRs were preferentially associated with intrinsic disorder. This finding supports the hypothesis that intrinsically disordered regions play a major role in the dynamics and diversity of TF networks through their ability to structurally adapt to and bind with multiple partners. Accordingly, this domain-based interaction resource represents an important step in refining protein interactions and networks at the domain level and in associating network analysis with biological structure and function.

## Introduction

Interactome networks are essential for complete systems-level descriptions of cells. Large-scale PPIs are integral in the analysis of topological and dynamic features of interactome networks [1], [2]. Several attempts to collect large-scale PPI data have been initiated using various model organisms [3], [4], [5], [6], [7], [8] and subsequently in humans [9], [10], [11]. Traditionally, protein interaction data are collected using high-throughput *in vivo* expression tools based on the yeast two hybrid (Y2H; [12]) and tandem affinity purification-mass spectrometry (TAP-MS; [13]) methods. Experiments of this nature have provided large-scale PPI data, but they have only generated information on interacting partners, without considering binding domains in detail. In the field of systems biology, a further understanding of cellular networks will require more complete data sets describing the underlying physical interactions between cellular components [14]. Thus, it is important to identify not only the binding partners, but also the interacting domain information at the amino acid level [14] (Supporting Data I in Text S1). In fact, the idea of mapping the interacting regions (IRs) involved in a PPI has been previously suggested for several large-scale screens [15], [16], [17], [18]. The mRNA display method of analyzing protein-protein interactions [19] developed in our laboratory is well suited to domain-based interactome mapping using a randomly primed cDNA library. The aim of this paper is to present the first human large-scale resource and mapping of IR data at the domain level for TF-related protein complexes using a high-throughput mRNA display screen. We believe that the results of this screen will lead to the improvement of network analyses.

**Figure 1:**
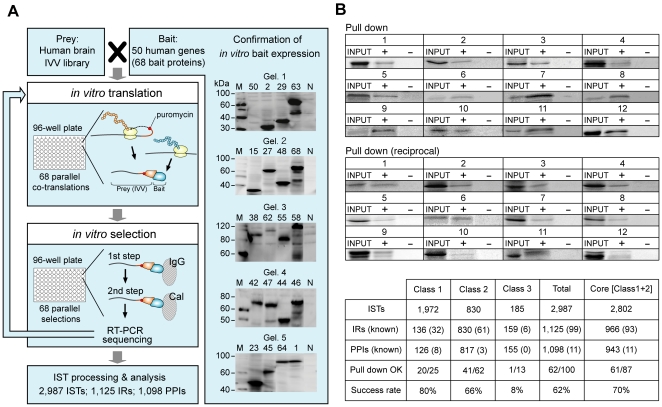
Toward the production of a comprehensive IR data set using IVV mRNA display technology. (A) Schematic of *in vitro* parallel auto-selection with IVV for large-scale analysis of PPIs and IRs. Individual steps (left) and expression of bait proteins (right) are indicated. This system is based on a modified high-throughput version of *in vitro* selection using IVV [19], consisting of four major steps (left side): (i) the preparation of mRNA templates that encode bait proteins and a randomly primed prey IVV library; (ii) *in vitro* parallel co-translation of bait and prey proteins and the formation of prey IVV as protein-RNA fusion molecules linked through puromycin and released from the ribosome; (iii) *in vitro* parallel selection, including RT-PCR and sequencing; and (iv) identification of PPIs and IRs by IST analysis (Figure S2). In IVV selection, ISTs are obtained as interaction fragmented sequences from a randomly primed prey IVV library. Bait protein expression was confirmed following *in vitro* translation by western blotting with an anti-T7 antibody (right side; ‘Confirmation of *in vitro* bait expression’). Lanes M and N indicate the molecular weight markers and negative control, respectively. Other lane numbers indicate bait protein IDs (Table S1). Expression was detected by 10–15% SDS-PAGE followed by protein staining. (B) Verification of PPIs (IRs) obtained following IVV selection by an *in vitro* C-terminal labeling pull-down assay [20]. Twelve representative examples of reciprocal pull-down assays are shown. ‘Pull down’ and ‘Pull down (reciprocal)’ indicate that assays were performed with the same and reciprocal combination of bait and prey (compared with the selection results), respectively. Binding was detected by 10–15% SDS-PAGE followed by protein staining. Also see Figure S4A. The bottom table summarizes the data obtained by the IVV selection approach for three classes (classes 1, 2, and 3; see also Supporting Data III in Text S1). The IVV core data set (Core) is defined as the proteins belonging to classes 1 and 2. The rows indicate the number of interaction sequence tags (ISTs), the number of interaction regions (IRs), the number of protein-protein interactions (PPIs), and the number of interactions verified by pull-down assay (pull down OK). The numbers of known PPIs overlapping with LC PPIs and known domains/motifs overlapping with the Pfam data [28] (Supporting Data V in Text S1) are given in parentheses.

To detect IRs at the domain level, we have performed a large-scale *in vitro* selection using *in vitro* virus (IVV; [19], [20]), a virus-typed protein-RNA fusion molecule, as a phenotype- and genotype-assignment molecule linked through puromycin [21] with a technique termed mRNA display [22], [23], [24], [25]. In this display technology, molecules that interact with target proteins are amplified by RT-PCR, and the amplified sequences are identified by DNA sequencing. Functional domains are easily extracted based on the identified sequences using a randomly primed prey library as a non-biased-representation [19], [26]. Bait mRNA templates were prepared using an *in vitro* procedure (Supporting Data II in Text S1 and Figure S1) that replaced the previous *in vivo* IVV cloning steps [19]. Large-scale mRNA display was performed using a biorobot that can simultaneously execute up to 96 selections. Because the modified IVV method is an entirely *in vitro* process, both toxic and nontoxic TF proteins can be characterized. This is a distinct advantage of this method because toxic proteins are not amenable to characterization by assays that require *in vivo* steps, such as Y2H [4], [9], [10] and TAP-MS ([5], [11]; Supporting Data I and II in Text S1). Fifty human TF-related proteins were used as bait, and a human brain cDNA library was used as prey. A modified high-throughput version of IVV selection was employed ([19]; Figure 1A).

Integration of large-scale PPI data with other data sets, such as 3D structural information [27] and expression data [2], is necessary to identify the possible functions of interaction networks [2], [27]. Large-scale IR data sets are expected to reflect functional domains and indicate the biological roles of the network without the need to integrate additional data. We confirmed the reliability and accuracy of our data by performing pull-down assays [19] and by examining the overlap between our results and known PPI domains with a Pfam search [28]. We subsequently conducted network analyses of TF-related complexes at both the protein and the IR levels. These analyses revealed that some IRs interact with multiple partners. Furthermore, we found that these IRs frequently include intrinsically disordered regions. This finding supports the hypothesis that intrinsically disordered regions, which may correspond to natively unstructured regions, play a major role in the dynamics and diversity of TF networks [29], [30], [31], [32], [33].

## Methods

### Modified Preparation of Bait mRNA Templates

We prepared 68 bait proteins representing 50 human TF-related proteins (Table S1). All 68 cDNA fragments (full length and/or domain portions of the TF-related proteins) were amplified by a four-step PCR with exTaq (Takara Bio) using a Qiagen Biorobot 8000. The PCR was performed as shown in Figure S1 and Tables S1, S2, S3. The mRNA templates were prepared with a RiboMAX Large Scale RNA Production System-SP6 (Promega) and m7G(5′)ppp(5′)G RNA Capping Analog (Invitrogen Corp., Carlsbad, CA, USA)[19]. The mRNAs were detected by routine western blot analysis using the anti-T7 antibody. Ninety-six percent of the bait proteins were expressed in the *in vitro* translation system using this method (Table S1). See ‘Supporting Methods’ (Text S1) for additional details.

### *In vitro* Parallel Auto-Selection Using IVV

A commercially available human brain cDNA library (the BioChain Institute, Inc.) was prepared for parallel auto-selection to be used as prey in large scale 96-well plate assays carried out by a Qiagen Biorobot 8000, according to a previously described method [19]. As directed by the reported method [20], a PEG Puro spacer was synthesized on a large scale by Invitrogen Japan K.K., Tokyo, Japan and Takara Bio Inc., Otsu, Japan. The human brain cDNA library to be used as prey was prepared according to a randomly primed cDNA library [19]. This approach reduces bias in the cDNA library. Moreover, interference from UTRs was not an issue in this system due to the use of an *in vitro* translation system. mRNA templates used as bait and prey were co-translated in a wheat germ extract (Zoegene Corporation, now Molecuence Corporation) for 1 h at 26°C in 96-well plates using a Qiagen Biorobot 8000. After six rounds of selection, the obtained sequences were identified by Takara Bio Inc., Otsu, Japan, and Shimadzu Corporation, Kyoto, Japan. A mock experiment was run without bait protein as a negative control to eliminate technical false positives in the IST analysis. See ‘Supporting Methods’ (Text S1) for additional details.

### IST Analysis

Determination of the interaction sequence tags (ISTs; Table S4) obtained from a randomly primed prey library was performed by Takara Bio Inc., Otsu, Japan and Shimadzu Corporation, Genomic Research Center, Kyoto, Japan. Using the IVV analysis system (IWAS)[19] developed by Fujitsu Limited, genes corresponding to each prey sequence were assigned by a BLASTN homology search against the coding sequences of the NCBI human RefSeq. Sequences with an E-value ≤1.0E-5 and a match length ≥30 bp were assigned as positive matches. Frame shift mutants were excluded from our analysis for the purpose of clarity. Finally, ISTs were classified into one of the following three categories: Class 1 ISTs were defined as those sequences overlapping with other prey sequence(s) obtained from the same bait protein (without distinguishing between partial and full-length proteins), excluding those that overlap prey sequences obtained from negative control experiments (mock experiments); Class 2 ISTs were defined as those ISTs that did not overlap with other prey sequences obtained from the same bait protein; and Class 3 ISTs were defined as the sequences that overlap with prey sequences obtained from mock experiments. Consequently, as shown in Figure S2, 1,972, 830, and 185, ISTs were obtained for classes 1, 2 and 3, respectively.

### Definition of Interacting Regions (IR) and Clusters

In order to identify important protein interaction regions (e.g., interacting domains, recognition motifs), we assessed the overlap among the IST regions of proteins obtained from common bait proteins. In the case of inclusive relationships between different overlapping regions, the innermost region was selected as the minimum length region. The selected regions were at least 3 amino acids in length. We defined a unique cluster as a group of ISTs sharing minimum length regions Moreover, we defined regions within the lateral ends of each cluster as maximum length regions, corresponding to an ‘interacting region’ (IR) containing the interacting domains and/or motifs (Figure S3 and Table S5).

### Verification of PPIs (IRs) by Pull-Down and Real-Time PCR Assays

In order to confirm interaction pairs (PPIs and IRs), pull-down experiments were performed as described in previous studies [19], [20], and the precipitates were analyzed by 10-17.5% SDS-PAGE. Real-time PCR was also performed to evaluate the interaction pairs. Briefly, each 20 µl reaction containing 5 ng of DNA template from the prey library obtained during each round of selection, gene-specific primers and SYBR Green PCR Master Mix (Applied Biosystems) was submitted to real-time PCR in a 7300 Real-Time PCR System (Applied Biosystems). Gene-specific primers corresponding to prey sequences were designed by Nihon Gene Research Laboratories.

### Pfam Search and Determination of Protein Contact Regions

**Figure 2:**
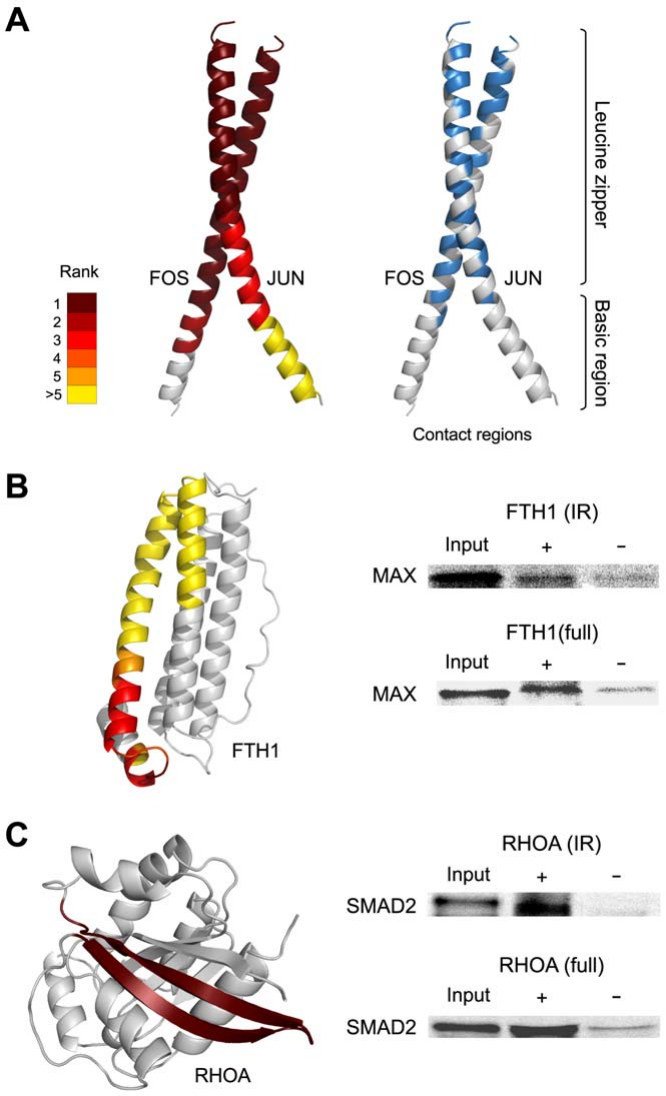
Validation of IR data obtained following IVV selection. (A) Left: IST density of IRs on the 3D protein structures of AP-1. ISTs obtained as prey in selections using FOS and JUN were mapped onto the 3D structure of AP-1 (PDB: 1A02, chain F and J; [55]. Right: Contact regions of AP-1. All amino acids of one protein within 4.0 Å of the other protein are colored blue. IST densities are ranked and colored on a scale of 1 to >5 according to the number of ISTs at each amino acid position. (B) Left: MAX interacting regions in FTH1. Twenty-four ISTs derived from FTH1, obtained using MAX as bait, were mapped onto the 3D structure of FTH1 (PDB:1FHA; [56]). Right: Pull-down assay to evaluate the MAX/FTH interaction. ‘IR’ and ‘full’ correspond to the assays performed with the IR (region: 124.176) and full-length FTH, respectively, as bait. Full-length MAX was used as prey. (C) Left: SMAD2 interacting regions in RHOA. An IST derived from RHOA, obtained using SMAD2 as bait, was mapped onto the 3-D structure of RHOA (PDB: 1OW3, chain B; [57]). Right: Pull-down assay to evaluate the SMAD2/RHOA interaction. ‘IR’ and ‘full’ correspond to the assays performed with the IR (region: 38..63) and full-length RHOA, respectively, as bait. The (522..1401) region of SMAD2 was used as prey.

Domains and motifs were identified through the following three steps: (1) BLAST search of each IVV sequence against the human RefSeq protein database; (2) extraction of the RefSeq protein fragment corresponding to the hit region; and (3) searching for the domains and motifs in each protein fragment. The “hmmpfam” was used to identify known protein domains and motifs in the Pfam database [28] (http://pfam.janelia.org/). Using all class 1 data that overlapped with Pfam domains, we demonstrated good alignment with the corresponding full-length proteins (Figure S5, S6 and Table S6). In order to determine the amino acids responsible for the interaction between two proteins, the distance between the main chain atoms of the two proteins was considered. We defined interacting amino acids as those amino acids possessing atoms within 4.0 Å of each other (Figure 2A). All 3D protein structures presented in this paper were created using PyMol (http://pymol.sourceforge.net).

### Mapping of the Transcription Factor (TF) Network at the Protein and IR Levels

**Figure 3:**
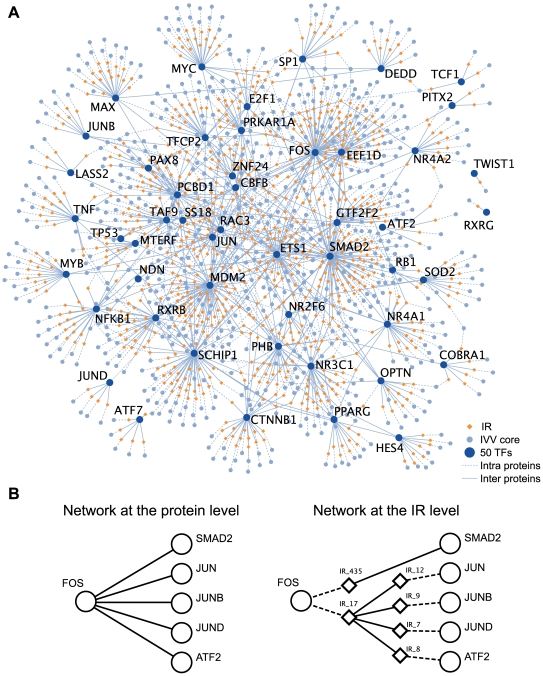
A TF network at the IR level developed using IVV data. (A) Graphic expression of the PPI network at the IR level. Interacting interfaces of the proteins, determined as IRs by IVV experiments, are drawn on the graph as diamond-shape nodes (IR nodes). Broken and solid lines indicate ‘intra-’ and ‘inter-’ protein edges, respectively. The graph contains 1,572 nodes (842 IR nodes and 730 protein nodes) and 842 intra-protein edges. Note that overlapping IRs are merged into a single node in the constructed network. Also see Figure S12. (B) An example of an underlying network graph at the IR level. Graphical expression of the FOS network at the protein level (upper). PPIs are simply expressed by nodes indicating proteins and edges that connect them. Graphical expression of the FOS network at the IR level (lower). A leucine zipper region of the FOS protein exclusively interacts with leucine zipper regions of other proteins (JUN, JUNB, JUND and ATF2). In addition, a region distinct from the leucine zipper in the FOS protein interacts with SMAD2.

The interaction network of human TF-related proteins was constructed by merging our IVV core data set and LC interactions (ftp://ftp.ncbi.nlm.nih.gov/gene/GeneRIF/interactions.gz) for the 50 TF-related proteins that were used as bait in our experiment (Figure S7 and Table S7). We also generated refined PPI networks based on IR data. An IR-level network graph is different from a classical PPI network graph because it contains intermediate nodes (i.e., IR nodes) between each interacting protein pair. All IR nodes have both intra- and inter-protein edges. Intra-protein edges reflect the fact that an IR is part of a protein. On the other hand, inter-protein edges represent interactions between different molecules (e.g., a bait protein and an IR). All of the network graphs were produced using Cytoscape [34]. Cytoscape files for Figures 3A and S12 are available upon request (contact E.M-S[nekoneko@educ.cc.keio.ac.jp]).

### Analyses of IRs with Multiple Interaction Partners

We defined IRs that shared 50% or more of their targets with other IRs obtained from different bait proteins as IRs with multiple partners. This class of overlapping IRs was unified as a single IR node in the network graphs. Proteins interacting with such IRs may compete with each other.

### Analyses of Intrinsically Disordered Regions

Intrinsically disordered regions of each human RefSeq were predicted using DISOPRED2 [30]. The default false positive rate (5%) of DISOPRED2 was used as a disorder/order classification threshold. Disordered regions in each IR were identified by comparing the positions of IRs and disordered regions assigned to the corresponding RefSeq. The proportion of disordered (or ordered) regions in each dataset (Table S11) was calculated as the total number of amino acid residues residing in disordered (or ordered) regions divided by the number of total amino acids in the dataset. The statistical significance of the differences between each group was determined using Fisher's exact probability test in R (http://www.R-project.org).

### Additional Methods

The core data set was analyzed for correlations with biological attributes, such as network properties (scale-free, etc.), expressional correlations, gene classification according to GO, and tissue-specific correlations. Descriptions of these network analyses are available in the Supporting Methods section (Text S1).

## Results

### Large-Scale IR Data Sets Obtained for 50 Human TF-Related Proteins by mRNA Display

For this large-scale mRNA display study, 68 bait proteins were prepared either from full-length proteins or from protein domains of 50 human TF-related proteins (Supporting Data II in Text S1). The display technique consisted of an *in vitro* parallel automated selection of IVV. The cell-free wheat germ translation system utilized in this study exhibited an excellent bait expression rate (96%) for human TF-related proteins. This system was modified from a previously published bait preparation [19] (Figure 1A, ‘Confirmation of *in vitro* bait expression’; also see Supporting Data II in Text S1, Figure S1 and Table S1). The success rate of the *in vitro* PPI selection for TFs was 99% (Table S1). These results suggest that this large-scale *in vitro* system provides highly effective protein expression and selection of TF proteins (Supporting Data II in Text S1). In the course of selection, we obtained 2,987 interaction sequence tags (ISTs), which were subjected to BLASTN searches to identify their corresponding proteins. The sequences were amplified by RT-PCR and sequenced (Figure 1A; Supporting Data III in Text S1). Of the 2,987 analyzed ISTs, 1,127 IRs were identified among the prey proteins (Supporting Data III in Text S1). The 2,987 ISTs (1,125 IRs and 1,098 PPIs) were subdivided into three classes (Figure 1B, bottom; see also Methods and Supporting Data III in Text S1). The IVV core data set (966 IRs; 943 PPIs for 730 proteins) was composed of classes 1 and 2. Class 3 ISTs (potential false-positives) were excluded from the dataset. However, data for classes 1 and 2 are presented (Table S4 and S5). The IVV core data are also available from the Genome Network Platform (http://genomenetwork.nig.ac.jp/index_e.html).

### Confirmation of the Core Data and Testing for False Positives

To confirm the reliability of the core data obtained by IVV parallel auto-selection, we carried out pull-down assays of 100 PPIs (IRs) using the C-terminal protein labeling method ([19], [20]; Figure 1B; Supporting Data IV in Text S1, Figure S4A, and Table S5). As expected, class 1 IRs displayed the highest confidence level (80%), likely due to the multiple ISTs for each prey protein. Further, class 3 IRs showed a much lower confidence level (8%), likely due to agreement with ISTs in a technical false positive data set in the negative control experiment (Figure 1B, bottom). Accordingly, we defined the IVV core dataset as all members of classes 1 and 2. The average verification rate for the IVV core data was 70% (Figure 1B, bottom). Because the pull-down assay is not a definitive verification experiment, real-time PCR [19] was used as an alternative assay to confirm the enrichment of prey genes in the IVV libraries. The results of the real-time PCR experiments were generally consistent with those of the pull-down assay (Supporting Data IV in Text S1 and Figure S4B). Together, these results, which are similar to those observed in large-scale Y2H experiments [9], [10], indicate that the large-scale *in vitro* experimental data are reliable and valid. Although the large mRNA moiety of IVV would likely interfere with protein interactions and *in vitro* folding would occur differently, we believe that interaction with and folding are less difficult to demonstrate for a protein domain than for a full-length protein. For this reason, the IVV library is composed mostly of parts of full-length proteins as a randomly primed prey library. Further, we previously attempted to assess the reliability of mRNA display both *in vitro* [19] and *in vivo* [26]. Experiments using ‘protein’ (not hybrid molecule) pull-down and co-immunoprecipitation assays demonstrated 80% accuracy, even in *in vivo* verifications. Note that the verification rate of pull-down assays obtained for the IVV method represents a minimum value because the method detects both direct and indirect interactions [19].

### Network Graph and False Negatives

The network graph (Supporting Data VI in Text S1 and Figure S7) shows the union of the IVV core data set (943 PPIs, 730 proteins) and the literature-curated (LC) data set for the 50 human TFs (1,240 PPIs, 796 proteins). This network contains 1,410 nodes (proteins) and 2,172 edges (PPIs; Table S7). The IVV core data set contains 82% more nodes than the LC data set (Table S8). It is of particular interest that we detected an average of 14 PPIs per bait protein in this IVV experiment, whereas previous Y2H experiments only detected an average of 3 PPIs per bait protein ([10], [35]; Supporting Data VI in Text S1). This difference suggests that Y2H might generate more false negative data than IVV in large-scale experiments. The difference between IVV and Y2H probably reflects the differences in the nature of the experiments (i.e., the difference between *in vitro* and *in vivo* binding behavior). The *in vitro* IVV selection system can utilize a larger library size and can also evaluate cytotoxic and self-activating TF proteins (Supporting Data I and II in Text S1). We suggest that the systematic collection of IR data from ISTs obtained with IVV, in addition to data collected by other methods such as Y2H, will be valuable for refining our understanding of protein interactions.

### Network Analyses of Biological Attribute

We found that the topological properties of the IVV core data network are similar to those of other interactome networks [9], [10]. Specifically, the IVV core data network shows a degree distribution that is approximately power-law degree, as well as a hierarchical organization and a tendency for highly connected (hub) proteins to interact with less highly connected proteins, as assessed by the degree distributions ([36]; Supporting Data VII in Text S1, Figure S8, and Table S9). In addition, we found that the interacting protein pairs identified by analysis of gene expression data for various cells and tissues using SymAtlas (http://wombat.gnf.org/downloads/GNF1Hdata.zip; [37]) were better correlated than would be expected by chance (random pairs; SupportingData VIII in Text S1 and Figure S9). These results suggest that selection of the prey library is very important for the generation of cellular networks. As we employed human TF proteins as bait, the data showed more frequent functional correlations with transcription-related terms in the Gene Ontology (GO) annotations (Supporting Data IX in Text S1 and Figure S10). Because a human brain cDNA library was used as prey, brain-specific PPIs were much more frequently detected in our experimental data than other tissue-specific PPIs (Supporting Data X in Text S1, Figure S11, and Table S10). Despite the fact that the IVV core data were generated in an *in vitro* experiment, it showed biological network properties (Supporting Data VII in Text S1, Figure S8, and Table S9) and biological expression correlations (Supporting Data VIII in Text S1 and Figure S9) similar to those found in previous large-scale *in vivo* experimental data sets [9], [10].

### Pfam Search and the Accuracy of IR Data As Functional Domains

Further analysis was performed to confirm the reliability and accuracy of IRs in the IVV core data set. A Pfam search ([28]; Supporting Data V in Text S1) was carried out to identify known domains within the IRs defined by IST analyses (Supporting Data III in Text S1). Based on this analysis, we identified 24% of the known domains (Figure 1B bottom, class 1, IRs (known)) within the class 1 data (136 IRs), indicating that Pfam domains are more concentrated in class 1 IRs than in class 2 IRs (Figure 1B bottom; Table S6). To confirm the accuracy of the IR data for FOS/JUN (activating protein-1; AP-1)-interacting domains aligned with ISTs (Figure S6), we compared the denser regions of IRs in the alignment of ISTs of FOS/JUN (Figure 2A, red and orange) with contact regions identified by the evaluation of 3D FOS/JUN structural data (Figure 2A, blue). We confirmed a precise agreement between the denser regions of IRs in the alignment of ISTs and the contact regions in the 3D structure data (Figure 2A, blue). Also, evaluation of all class 1 data that overlap with Pfam domains demonstrated good alignment with corresponding full-length proteins (Figure S6), demonstrating the reliability of the IR data (Figure 2A). Thus, the IR data provide reliable and accurate information about binding interfaces (functional domains) involved in protein interactions (Supporting Data V in Text S1).

Pull-down assays were employed to validate the IRs within Pfam domains. Figs. 2B and C show representative assays. Proteins evaluated by pull-down were selected from both class 1 and class 2. We identified MAX/FTH1 from class 1 and SMAD2/RHOA from class 2 as PPI pairs with well-conserved domains (Table S6). MAX and FTH1 contain helix-loop-helix (HLH) and ferritin domains, respectively (Table S6). SMAD2 and RHOA contain MH2 and Ras domains, respectively (Table S6). We reciprocally confirmed PPIs for both MAX/FTH1 (Figure 2B) and SMAD2/RHOA (Figure 2C) with C-terminal labeling pull-down assays using both protein domains and full-length proteins. The domain-domain interaction between HLH and ferritin has also been identified in *Drosophila melanogaster* (1 pair [6]). In addition, the interaction between MH2 and Ras has also been observed in other PPIs in humans (47 pairs [38] and an additional SMAD2/RAN pair in the IVV core data; Table S6) and other species (3 pairs [7]). Accordingly, it is likely that IR data will allow for the prediction of PPIs and domain-domain interactions (DDIs) based on domain information [39], [40]. We verified two interaction domains identified by an IVV experiment, including a globular domain in which the interaction region is clearly distinguished from the rest of the protein (Figure 2C).

### Network Graph at the IR Level

In order to provide an overview of our experimental results, we drew a refined interaction network graph at the IR level containing 1,572 nodes (842 IR nodes and 730 protein nodes) and 842 intra-protein edges (Figure 3A; Figure S12). Note that the overlapping IRs are merged into a single node in the network diagram. We have derived an example (FOS) of a network graph at the protein level (Figure 3B, left) and the IR level (Figure 3B, right). The underlying network graph of FOS at the IR level shows two different interactions (two different IR nodes), AP-1 (FOS/JUN) and FOS/SMAD2 (Figure 3B, right), which is analogous to the well-known AP-1/Smad3 complex [41], [42], [43]. The interaction between FOS and JUN is also well known [44]. We have confirmed the newly identified interaction between FOS and SMAD2 via a pull-down assay using the C-terminal protein labeling method (data not shown). FOS contains an IR (IR_17) that competitively interacts with multiple partners (JUN, JUNB, JUND, or ATF2), as well as an IR (IR_435) that exclusively interacts with one partner (Figure 3B, right). It is important to distinguish between interfaces with competitive and non-competitive properties to understand the dynamics of cellular networks in detail [27]. Thus, once we obtain an underlying network graph at the IR level (Figure 3B, right) instead of a classical network graph at the protein level (Figure 3B, left), we can identify refined interaction network interfaces and the biological implications of those results for TF complexes.

### Network Analysis of Intrinsic Disorder Rate at the IR Level

**Figure 4:**
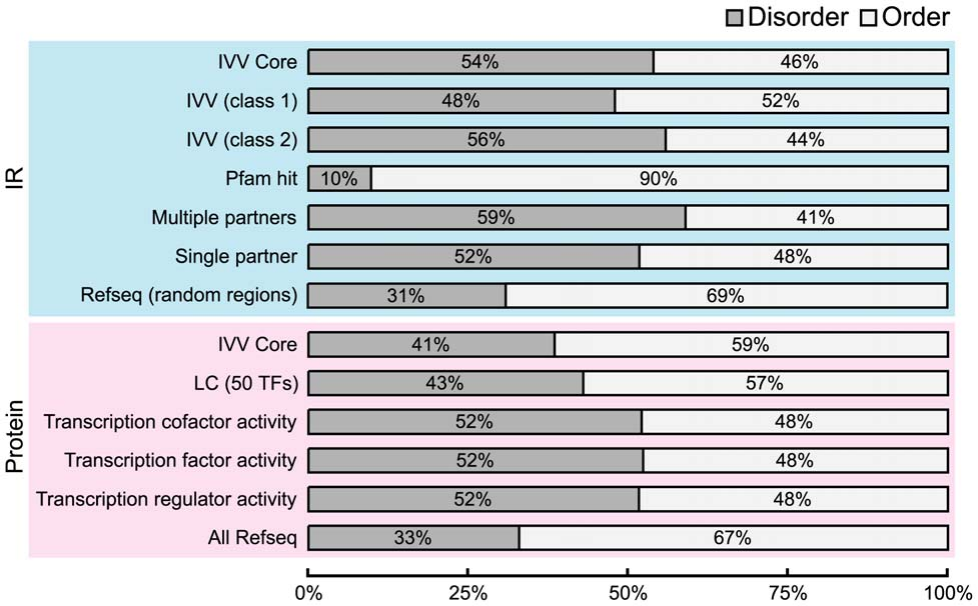
Analysis of the rates of disordered regions. The proportions of intrinsically ordered and disordered regions in 13 datasets consisting of IR (7 datasets) and Protein (6 datasets) were analyzed by DISOPRED2 [30] as follows: IR (IR-level data); IVV Core; IVV (class 1); IVV (class 2); Pfam hit (a set of IRs hit by Pfam search); Multiple partners (IRs obtained from multiple bait proteins); Single partners (IRs obtained from a single bait protein) and Refseq (random regions) or Proteins (protein-level data); IVV Core; LC (a set of known interacting partners for 50 bait proteins); ‘Transcription regulator activity’ (a set of proteins for which GO:0030528 is assigned); ‘Transcription cofactor activity’ (a set of proteins for which the GO:0003712 is assigned); ‘Transcription factor activity’ (a set of proteins for which GO:0003700 is assigned); and All RefSeq: all human RefSeqs. The dataset of random regions was created by random selection of protein regions (n = 10000) from the human RefSeq that together correspond to the same length distribution as that of detected IRs. Information about the assignment of GO identifiers for proteins can be obtained from the Gene Ontology Web site (http://www.geneontology.org).

Following Fischer's lock-and-key proposal, many counterexample proteins have been identified that require a lack of three-dimensional structure in order to function. The importance of the lack of three-dimensional structure (related to disordered regions) in protein interactions can no longer be ignored [29]. We examined the rates of ordered/disordered regions [29], [31] in the IVV core data set (943 PPIs; 966 IRs) using DISOPRED2 [30] in order to obtain a detailed understanding of the types of structural interactions of human TF complexes (Supporting Data XI in Text S1 and Table S11). Figure 4 shows the proportions of intrinsically disordered regions (residues) in various groups of IRs or proteins. The proportion of disordered regions (residues) in the IVV core data was significantly higher than that in the human Refseq for both regions and protein levels (p<2.2e–16 in each comparison); a more distinctive difference was observed for IRs (Figure 4, IVV core (IR)). A similar trend was observed in three previously reported transcription factor data sets [45]. In a detailed comparison within the IVV core data set, we observed more disordered regions (residues) in class 2 IRs than in class 1 IRs (p<2.0e-06). In addition, IRs identified by the Pfam search program showed a higher proportion of ordered structures than any other set of IRs (p<2.0e-06 in every comparison). Prey proteins, which bind to bait proteins with higher affinity, are expected to be in class 1, a group of IRs consisting of multi-targeted prey sequences. In principle, since this experiment is based on affinity selection, stronger binding to bait proteins should correspond to a higher probability of detection. In addition, a wider variety of proteins (genes) are categorized as class 2 (830 proteins, Figure 1B, bottom) than class 1 (136 genes, Figure 1B, bottom) when the 50 human TFs are used as bait. Taken together with the frequent observation of disordered regions in class 2 proteins, these results indicate that many interacting partners of the 50 TFs appear to display unstable interactions mediated through their disordered regions. A limited number of partners were identified with stable interactions involving ordered regions. In the *in vivo* situation, various combinations of interactions could occur depending on the physiological context (location, time, etc.). Thus, we consider that disordered regions not only of TFs, but also those of the interacting partners of TFs are employed as interaction interfaces to achieve the dynamics necessary for formation of diverse TF complexes. These findings suggest that IVV technology can identify both stable and less stable interactions involving disordered regions. The current understanding regarding the affinities of binding mediated by disordered regions is that these affinities are weak in terms of entropy [46]. In fact, many low-affinity (flexible) interactions are included in the IVV core data set, and utilization of the IVV method almost doubles the size of the identified interactome network (the IVV method produced 82% more nodes than did the LC data set (Table S8)). On the other hand, a relatively low percentage of known domains in the IVV core data set (10%, Figure 1B, bottom) are derived from the quantitative dominance of low-affinity interactions.

**Figure 5:**
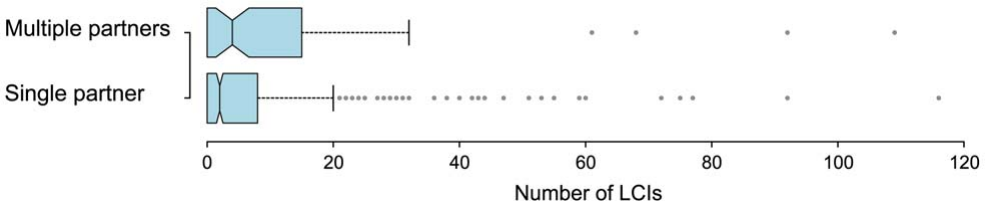
IR properties and the number of known interaction partners. Counts of LCI for each prey gene (protein) were plotted for two datasets: prey proteins having IRs obtained from multiple bait proteins (multiple partners), and proteins having IRs obtained from a single bait protein (single partner).

We further examined the characteristics of IRs displaying multiple interaction partners in the IVV experiment (Figure 3). We speculate that these multi-targeted IRs interact with numerous other partners in a cellular context. Figure 5 shows plots of the number of interaction partners for each prey protein. The mean number of known interaction partners for proteins containing multiple interacting IRs was 11.1, significantly higher than the mean for other prey proteins of 6.8 (Wilcoxon rank-sum test, p = 0.003). In addition, the proportion of disordered regions (residues) in IRs with multiple partners was 59%, significantly higher than that for any other dataset in our analysis (Figure 4; p<0.0001 for every comparison). These findings indicate that disordered IRs can provide the ability to interact with multiple different proteins. This assumption is consistent with the results of several previous studies on the potential of disordered regions frequently observed in so-called hub proteins [32], [47], [48], [49], [50]. In addition, this finding supports the hypothesis that transcriptional regulatory proteins frequently bind to various partners [51]. The IRs with multiple partners detected in this experiment might also function as flexible interfaces that mediate interactions among various compatible partners.

## Discussion

In order to obtain a large-scale IR data set that covers the huge interactome space, we conducted a novel large-scale, automated *in vitro* experiment using an mRNA display methodology (Figure 1A). This large-scale *in vitro* strategy is not subject to difficulties in protein expression (the TF expression rate was 96%) because living cells are not used. The core data set generated by this experiment showed a verification rate of at least 70%, similar to that of the more traditional Y2H approach [9]. The IVV mRNA display method uncovered the interactome network more efficiently (14 PPIs per bait protein) than the Y2H method (3 PPIs per bait protein [10], [35]). Several indirect interactions [19] were detected by the IVV method, although they could not be verified. However, their presence in the data set suggests that the IVV method has the same or a lower rate of false positive and false negative identification as the Y2H approach. The core data set suggested biological attributes similar to those identified by previous large-scale *in vivo* experimental data sets [9], [10]. In particular, the nature of the expression correlation (Supporting Data VIII in Text S1 and Figure S9) suggests that selection of the prey library is very important for uncovering cellular networks. Accordingly, we have developed an automated large-scale analysis tool suitable for collecting not only PPI but also IR information over the human protein interactome space of nearly 300,000 PPIs [52].

Importantly, this work has yielded not only a large-scale data set of interaction partners, but also the first large-scale resource of human IR data obtained by IVV; this data set links network analysis and biological understanding [14]. The IVV prey library, prepared by means of a random priming method, contains randomly primed sequences encoding parts of proteins. This approach allows for analysis of interaction domains and reduces bias in the cDNA library, such as the bias toward the 3′ ends of mRNA, as was the case in the identification of *C. elegans* domain-based interactions by Y2H [18]. This study represents the first use of a high-throughput version of mRNA display to map large-scale domain-based interactions, especially for human TF-related proteins. The refined domain-based IR-level network graph (Figure 3) and the corresponding functional domains (Figure 2) reveal characteristic competitive or non-competitive interactions in the human TF network (Figure 3). Further, the network suggests that human TFs preferentially interact with disordered regions (Figure 4). In particular, proteins capable of interacting with multiple partners through the same IRs showed the greatest disorder (flexibility) (Figure 4), can act as network hubs (Figure 5), and may correspond to disordered regions that play a crucial role in determining the dynamics and diversity of transcription regulatory networks [51]. However, it is still unclear whether disordered IRs are a specific to interacting partners of TF-related proteins. Further experiments must address this issue via comparisons using other bait proteins which are not related to TFs.

In addition, large-scale IR data can lead to the identification of functional domains (Fig. 2; Figure S6 and Table S6), allowing for the prediction of PPIs and domain-domain interactions (DDIs) [39], [40] in the interactome space ([52], [53]; Supporting Data I in Text S1). Large-scale disordered IR data will be helpful in reassessing the traditional structure-function paradigm (the lock-and-key hypothesis; [29], [31]). In addition, this type of data may be useful for protein crystallization studies in structural genomics projects ([33]; Supporting Data XI in Text S1) because it is easier to investigate the structures of such disordered regions by X-ray crystallography or nuclear magnetic resonance (NMR) spectroscopy in the presence of interaction partners (protein domains or full-length proteins) [31]. Furthermore, there is a high level of interest in targeting the interfaces between interacting proteins for therapeutic purposes [54] (Supporting Data XI in Text S1). The identification of interface sequences may also help in the *de novo* design of functional proteins and peptides. Once the IR data are obtained, mutations, SNPs, spliced exons, and sites of post-translational modification within IRs may be analyzed. Although this IR data set is far from complete, even for human TF complexes, we believe that the systematic collection of IR data from ISTs obtained by IVV, Y2H, and other methods will be valuable for refining protein interactions, enabling us to understand cellular events in greater detail.

## Supporting Information

Text S1 Supporting data and methods.
(0.26 MB DOC)

Figure S1 Strategy of mRNA preparation for bait proteins. Primer 1 consists of a gene-specific sequence (sky-blue box) and the T7 tag (yellow box) sequence. Primer 2 consists of a gene-specific sequence and part of the affinity tag sequence (green box). These primers were used to connect a gene with tag sequences. The tagged construct was amplified by primers 3 and 4. Primer 5 consists of the promoter (orange box) and the T7 tag sequence. Primers 4 and 5 were used to connect a gene to a promoter. A bait protein encoding mRNA was then transcribed from the 4th PCR product.
(0.04 MB PDF)

Figure S2 Flow chart of interacting sequence tag (IST) analysis after IVV selection. ISTs of prey proteins were detected, evaluated for quality of alignment to reference sequences of human genes and subdivided into 3 classes. See ‘IST analysis’ in the Supporting Methods section.
(0.03 MB PDF)

Figure S3 Definition of interacting regions (IR) and clusters. An example of interacting regions (IRs) determined by five different ISTs (indicated by the shaded boxes) is shown. In the presented case, there are three clusters containing the minimum/maximum regions for each IR. The maximum regions correspond to IRs containing interacting domains and/or motifs. Colors (red, blue, purple) correspond to each cluster.
(0.33 MB TIF)

Figure S4 Verification of IVV PPIs (IRs) by pull-down and real-time PCR assays. (A) Results of the in vitro pull-down assay. Each pull-down assay number corresponds to a number in Table S5. Prey protein prior to elution (INPUT) and the eluate in the presence (+) and absence (−) of the bait protein are shown. (B) Real-time PCR results. The numbers correspond to the verification numbers (Table S5). The x-axis value indicates the round of selection and the y-axis value indicates the measured DNA copy number. Blue and red colors indicate the selection results with and without bait protein, respectively.
(0.60 MB PDF)

Figure S5 Procedure for identification of known protein domains/motifs in IVV IRs by a Pfam search with “hmmpfam.” Motifs were identified using the following 3 steps: (1) A BLAST search of each IVV sequence against the human RefSeq protein database; (2) Extraction of the RefSeq protein fragment corresponding to a hit region; and (3) Searching for the motif(s) in each protein fragment. The “hmmpfam” was used to find known protein motifs in the Pfam database.
(0.15 MB TIF)

Figure S6 Alignment of ISTs with Pfam domains. IST-mapped regions that overlapped with any Pfam domain/motif region were aligned with the corresponding full-length proteins. ISTs and Pfam domain/motif regions in the full-length proteins are represented by solid black and green squares, respectively. Only ISTs belonging to class 1 are illustrated in the figure. Pfam domain/motif regions were obtained from the Pfam ftp site (ftp://ftp.sanger.ac.uk/pub/databases/Pfam/current_release/swisspfam.gz). The following 31 bait/prey combinations are depicted: EEF1D/EPRS, ETS1/JUN, FOS/ATF2, FOS/CABP1, FOS/JUN, FOS/JUND, FOS/HSPA1A, JUN/ATF2, JUN/CREB3, JUN/FOS, JUN/HSPA8, JUN/MAPRE3, MAX/FTH1, MAX/FUS, MAX/RPL34, MAX/RPL35, MAX/TUBA3, MDM2/APP, MDM2/CLU, MDM2/JUN, MDM2/JUND, MDM2/PKM2, MYC/KIDINS220, PAX8/ANXA7, PHB/COX6C, SCHIP1/TMSB4X, SP1/NAP1L1, SP1/TPI1, SMAD2/JUN, TAF9/FEZ1, TAF9/RPS24.
(0.83 MB PDF)

Figure S7 PPI network focused on 50 human TF proteins. A merged network of IVV core data and LC PPI data focused on 50 human TFs. Nodes corresponding to the 50 TFs are indicated in blue. LC PPIs are indicated by black edges and white nodes in the graph. Newly identified PPIs are indicated by red edges and green nodes (see Data VI).
(2.61 MB TIF)

Figure S8 Degree distributions. (A) Degree distribution of the nodes in the PPI network generated from IVV data; (B) Degree distribution of the nodes in the network generated from LC data on PPIs directly related to the 50 TF proteins used as bait in the IVV experiments.
(0.27 MB TIF)

Figure S9 Expression correlations of PPIs obtained with the IVV method. The horizontal and vertical axes show expression correlations of interacting pairs and their rates among all of the pairs, respectively.
(0.13 MB TIF)

Figure S10 Gene classification by Gene Ontology (GO). The frequencies of the GO terms from the following five data sets are shown: (1) Human proteome (http://cvsweb.geneontology.org/cgi-bin/cvsweb.cgi/go/gene-associations/gene_association.goa_human.gz?rev=HEAD), IVV (Core), (2) the dataset limited to prey genes (proteins) belonging to class 1; (3) the dataset limited to prey genes (proteins) having any motif/domain in the IST regions; (4) the Y2H data set including genes (proteins) obtained as the prey; and (5) the Y2H; TF) data set limited to the prey genes (proteins) that interact with baits having GO assignments of ‘transcription regulator activity’ or ‘transcription factor activity.’ GO identifiers for genes in each data set were counted in three main categories of ontology: A, ‘Molecular function;’ B, ‘Biological process;’ and C, ‘Cellular component.’ GO slim files (http://www.geneontology.org/GO_slims/goslim_generic.obo) were used to summarize annotations for each data set.
(1.22 MB TIF)

Figure S11 Tissue-specific PPIs. The histogram shows the proportions of tissue-specific PPIs (Data X) in the possible analytical space C, which is defined as the product of the number of tissue-specific genes and the number of bait proteins: 45,200 (904×50) in brain tissue; 25,300 (506×50) in liver tissue; 24,800 (496×50) in lung tissue; 14,050 (281×50) in kidney tissue; and 13,550 (271×50) in heart tissue. The numbers of brain-, liver-, lung-, kidney, and heart-specific PPIs, Nspecific, were 128, 10, 7, 5, and 4, respectively.
(0.05 MB TIF)

Figure S12 IR-level PPI network of 50 human TF proteins. A merged network of the IR-level PPI network of the IVV core data set and the LC PPI data set (1,240 LC PPIs) focused on 50 human TFs. Nodes corresponding to the 50 TFs are indicated in blue. Interactions from the IVV and LC data sets are indicated by red and blue edges in the graph, respectively (see Figure 3A). All of the network graphs were produced in Cytoscape. Cytoscape files (IVV_IR_Networks.cys'), including this figure and Figure 3A, are available upon request (contact EM-S[nekoneko@educ.cc.keio.ac.jp]).
(2.82 MB TIF)

Table S1 List of bait proteins. Entrez Gene IDs, Official Symbols and GenBank accession numbers of bait proteins are shown. Positions indicate the region of the sequence corresponding to the accession number. ‘BasePair’ and ‘Weight’ indicate the length and molecular weight of the bait protein. Primers 1 to 5 are the primer names (see Table S2). The program names refer to the PCR programs (see Table S3). NG in the ‘Selection result’ column indicates that the bait protein obtained no prey interactors. An asterisk indicates that the bait protein cDNA was prepared as described previously. Although the 2nd PCR was normally performed with the 5′baitCBP and 3′FosCBPzz primers (See ‘Preparation of bait mRNA templates’ in Supporting Methods), construction of ProteinID 60 with a 3′ deletion required an additional 2nd PCR step using the 5′TAF9_2ND_012B and 3′FosCBPzz primers with PCR program #1 for preparation of the full-length template (see Figure S1).
(0.05 MB XLS)

Table S2 List of primers used in the preparation of bait protein cDNAs (see also Figure S1 and Tables S1 and S3).
(0.05 MB PDF)

Table S3 List of PCR programs used for amplification of bait cDNA templates.
(0.12 MB PDF)

Table S4 List of interacting sequence tags (ISTs). The definition of ‘class’ is given in Figures S2 and S3 in the sections ‘IST analysis’ and ‘Definition of interacting regions (IR) and clusters’ in the Supporting Methods. Data on class 3 are available upon request (contact EM-S [nekoneko@educ.cc.keio.ac.jp]).
(0.67 MB XLS)

Table S5 List of interacting regions (IRs) and verifications. ‘IR’ and ’Domain/motif (Prey)’ show IRs of prey proteins and the results of ‘Pfam searches’, respectively. ‘Verification IST number’ corresponds to the IST number used in the verification assays. See also Figures S3 and S4, ‘Definition of interacting regions (IR) and clusters’ in Supporting Methods. ‘Known PPIs’ indicate PPIs that overlap with LC PPIs obtained from NCBI (12/18/2006 ftp://ftp.ncbi.nih.gov/gene/GeneRIF/interactions.gz). ‘Pulldown (reciprocal)’ refers to the results of reciprocally performed pull-down assays. See Figure 1B and the ‘Verification of PPIs (IRs) by pull-down and real-time PCR assays’ section in the Supporting Methods. Data on class 3 are available upon request (contact EM-S [nekoneko@educ.cc.keio.ac.jp]).
(0.22 MB XLS)

Table S6 List of Pfam domains assigned to IRs.
(0.04 MB XLS)

Table S7 List of interacting protein pairs related to the 50 human TFs.
(0.25 MB XLS)

Table S8 Increase of LC PPIs by the IVV and Y2H data sets.
(0.05 MB PDF)

Table S9 Comparison of the network characteristics.
(0.06 MB PDF)

Table S10 Frequencies of tissue-specific PPIs in the IVV core data set.
(0.04 MB PDF)

Table S11 Proportions of ordered/disordered regions in each IR.
(0.20 MB XLS)
