# Supplementary material for: A Comprehensive Resource of Interacting Protein Regions for Refining Human Transcription Factor Networks
Source: PLoS One. 2010 Feb 24;5(2):e9289. doi: 10.1371/journal.pone.0009289 (PMC2827538; doi:10.1371/journal.pone.0009289)
Supplement: Text S1 — Supporting data and methods. (0.26 MB DOC) [file pone.0009289.s001.doc]

# Supporting Information

# Supporting Data

**I. Large-scale mRNA display for refining protein interaction networks**

Many previous studies [1,2,3,4,5,6,7,8,9] have reported large-scale protein-protein interaction (PPI) data and explored the global aspects of network topology. These interaction networks have been linked to protein function, expression dynamics, and other genomic features. However, most network studies have not considered binding domains or motifs in detail. In the area of systems biology, progress in understanding cellular networks will require more complete data sets that describe the underlying protein interactions [10]. The purpose of this paper is to present a first step in the collection of large-scale interactions at the domain level using interacting region (IR) data for human TF complexes. We use an *in vitro* selection methodology [11] to avoid problems implicit in *in vivo* experiments [12,13]. We expect that refinement of protein interaction data sets using large-scale IR data may facilitate predictions of domain-domain interactions (DDIs) and PPIs [14]. Therefore, IRs derived from ISTs should yield new insights, not only for analyses of protein interaction partners, but also for analyses of detailed cellular networks with implications for the dynamic features of complexes.

Alternative approaches for large-scale experiments are required to obtain large-scale IR data that cover the huge interactome space and to offset weaknesses of *in vivo* methods. This paper reports the use of mRNA display technology on a large scale for the first time. We employed a modified high-throughput version of an *in vitro* virus (IVV; [11]). *In vitro* selection, based on a characteristic property of puromycin [15], has been developed as an mRNA display technology [16,17] and applied for the detection of protein interactions [11,18,19,20,21]. The IVV method can extract functional domains involved in PPIs from a very large library in a single experiment. Also, the modified IVV method is an entirely *in vitro* manipulation, unlike other methods such as Y2H [2,4,5,7,8], phage display [22], and protein purification-mass spectrometry methods such as TAP-MS [3,23]. Since our method does not include any *in vivo* steps, it allows for the evaluation of toxic proteins.

# II. Preparation of bait proteins for IVV parallel auto-selection of 50 human TFs

We modified the preparation of bait proteins used in the high-throughput version of IVV ([11]; Figure S1) and prepared 68 bait proteins related to 50 human TF proteins, including 38 TFs and 12 transcription-related proteins for which clones are commercially available (as of 01/31/2005 Toyobo, 02/01/2005 Funakoshi and 02/02/2005 Invitrogen Corp., Carlsbad, CA, USA). To prepare multiple bait proteins for IVV parallel auto-selection on a Qiagen Biorobot 8000 (Table S1-3), the preparation of bait mRNA templates was modified so as to require only four PCR steps and one *in vitro* transcription step, in contrast to the previous time-consuming manual steps and cloning [11]. In total, 68 bait mRNA templates encoding 50 human TF proteins (Table S1) were prepared and expressed using a cell-free wheat germ translation system. Proteins were detected by western blot analysis using an anti-T7 antibody. All PCR, transcription, and selection steps were automated using a Qiagen Biorobot 8000. The expression rate of bait proteins was 96% and the selection success rate of bait proteins was 99% (Table S1). In a previously reported large-scale Y2H experiment, the success rate of nuclear receptors was only 79% [25]. These results suggest that our large-scale *in vitro* strategy does not encounter any problems with protein expression or self-activation [24] for TFs, unlike Y2H experiments that use living cells.

**III. Analyses of ISTs, PPIs, and IRs obtained from mRNA display**

To conduct a large-scale analysis of PPIs using the modified high-throughput version of IVV [11], a parallel selection with 68 bait proteins in 96-well plates was conducted by a Qiagen Biorobot 8000 (Figure 1A). We carried out mock experiments [11] as negative controls (without bait) to identify false positives and positive control experiments to monitor selection enrichment. In this experiment, sufficient enrichment was reached by the 6^th^ round of selection. Cloning and sequencing identified 2,987 interacting sequence tags (ISTs) and 1,098 PPIs using the IVV analysis system (IWAS; [11]) developed by Fujitsu Limited. Genes corresponding to each prey sequence were assigned through a BLASTN homology search against coding sequences of the NCBI human RefSeq (as of December 18, 2006). Those RefSeq entries with an E-value of ≤1.0E-5 and a match length of ≥30 bp were considered positive matches. We subdivided the ISTs into three classes (Figure S2). Class 1 ISTs were defined as those that overlapped with other prey sequence(s) obtained from the same bait protein (without distinguishing between partial and full-length proteins), excluding those that overlapped prey sequences obtained from negative control experiments (mock experiments); Class 2 ISTs were defined as those having no overlap with prey sequences obtained from the same bait protein; and Class 3 ISTs were defined as those overlapping with prey sequences obtained from mock experiments. Ultimately, we obtained 1,972, 830, and 185 ISTs for classes 1, 2 and 3, respectively. We analyzed IRs, defined as the maximum length regions containing interacting domains or motifs in prey proteins (Figure S3 and Table S5). We obtained 1,125 IRs, including 136, 830, and 159 IRs from classes 1, 2 and 3, respectively (Figure 1B and Table S5).

**IV.** **Confirmation of PPI (IR) data**

To verify the IVV selection data, 100 randomly selected PPIs (IRs) were confirmed using pull-down assays. Pull-down assays were performed using C-terminally fluorescently labeled prey proteins [11]. We obtained success rates of 80%, 66% and 8% for classes 1, 2 and 3, respectively (Figure 1B and Figure S4A). Because the class 3 data set was composed of technical false positives, we defined the IVV core data set as all members of classes 1 and 2. This core data set displayed a selection success rate of 70%, similar to the success rate for PPIs screened by Y2H. This indicates that the efficiency of IVV selection is comparable to that of Y2H screening. Because a pull-down assay is not a definitive verification experiment, real-time PCR [11] was performed to confirm selection enrichment for 22 of the 100 interaction pairs tested by pull-down assays (Figure S4B). These pairs were chosen because they were the only pairs for which primers could be designed. The results of the real-time PCR assays (19 for IVV core data) were consistent with those of the pull-down assays for all interaction pairs except for three (Table S5). Those three PPIs showed positive results in the real-time PCR assay but negative results in the pull-down assay. The negative results of the pull-down assay may indicate that these three interactions were indirect, because IVV selection is not limited to direct interactions [11]; it may also reflect the limited sensitivity of single experiment verification.

**V.** **Pfam search and accuracy of IR data**

A Pfam search (<http://pfam.janelia.org/>) [26] was conducted in order to identify known domains among the IRs (Figure S5, S6 and Table S6). We found 10% of the known domains in the core data (966 IRs) and 24% in the class 1 data (136 IRs; Figure 1B, bottom), suggesting that IRs probably represent important binding domains or motif regions of prey proteins. The ISTs of class 1 data that overlap with known domains (32 IRs) were well aligned with the corresponding full-length proteins, including FOS and JUN (Figure 2A; see also Figure S6). The FOS/JUN heterodimer [27] activator protein-1 (AP-1; [27]) is a well-known TF complex, and its co-crystallized structure has been elucidated [28]. Several ISTs derived from FOS and JUN were reciprocally obtained from each bait (Figure 2A). The AP-1 complex was the only available co-crystallized structure in which ISTs derived from the same complex could be simultaneously mapped. Multiple ISTs were mapped onto the 3D structure of the FOS/JUN heterodimer [27] and denser regions were colored red (Figure 2B, left). We then compared the mapped ISTs as IRs on the structure with all amino acids of FOS within 4.0 Å of JUN and *vice versa* (Figure 2B, right). Structural contact regions (FOS: 159..191aa/ JUN: 273..305aa; Figure 2B, red and orange) corresponding to the leucine-zipper motif (Pfam L-Zip domain [FOS: 136..189aa/ JUN: 250..314aa]) matched well with the IRs (FOS: 149..266aa/ JUN: 257..327aa; Figure 2B, blue), and only a few ISTs overlapped with the basic regions required for DNA binding (Figure 2B, left).

# VI. Transcription factor (TF) network at the protein level

Fifty TF proteins were used as bait in this experiment and the following analysis. At the time of this analysis, 1,240 literature-curated (LC) PPIs (796 proteins) were contained in the NCBI database (originally from HPRD and BIND) as interactions involving these 50 TF proteins. The IVV core data set consisted of 943 PPIs (730 proteins). Merging these two data sets resulted in a PPI network consisting of 2,172 PPIs (1,410 proteins; Figure S7 and Table S7). We investigated the number of interactions and proteins that were new additions to this merged PPI network by our experiment.

Expansion rates of the LC data by adding the new data that we obtained were 75% and 82% for PPIs and proteins, respectively (Table S8). The Y2H network was analyzed in the same way [8]. For TFs alone as bait proteins (116 TFs), the ratio of newly added PPIs and proteins to those of the LC data set was 0.48 and 0.32, respectively (Table S8). The difference between the IVV and Y2H data sets probably reflects the differences in the nature of the experiments (e.g., the differences between *in vitro* and *in vivo* protein expression and binding)*.* The *in vitro* IVV selection system can deal with cytotoxic proteins and self-activation of TF proteins, problematic issues for Y2H experiments (see also Data I and II).

We obtained an average of 14 reliable interactors (943 PPIs/68 baits) per bait, whereas the average number of interactors for the Y2H data set was only 3 interactors per bait (376 PPIs/116 baits) [8,29]. One possible reason for this difference is the fact that the *in vitro* system can deal with cytotoxic proteins and self-activation of genes of transcription-related proteins (see also Data I and II). Another possible explanation is that the *in vitro* system can utilize a larger library size (10^12-13^) and therefore should be capable of detecting more interactors in a single experiment. However, the problem of false negatives contained in the large library remains, as the library size is beyond the feasible capacity for sequencing analysis [11].

**VII. Network characteristics of interacting proteins obtained from IVV**

PPI networks generated from high-throughput experimental data such as Y2H data have been reported to exhibit scale-free network characteristics [7,8]. In the scale-free network, the distribution of the proportion of nodes with *k* edges (degree=*k*), *P*(*k*), follows an exponential (r) distribution (i.e., *P*(*k*)∝*k^-r^*). In a Y2H network, *r* has been calculated as 1.65 (*R*^2^=0.91)[8]. The value of *r* for the IVV network presented in this paper was calculated as 1.21 (*R*^2^=0.72), indicating that the distribution agrees with the power-law distribution (Figure S8A and Table S9). Although the IVV network showed a relatively small *r* exponent compared with the Y2H network, the *r* value for the network consisting of LC PPIs directly related to the 50 TF proteins used as bait was 1.07 with *R*^2^=0.73 (Figure S8B and Table S9). This indicates that the value of *r* in the IVV network was affected by the relatively small analytical space (limited to 50 TF proteins).

Hierarchical properties are shared within various complex networks. The distribution of the mean clustering coefficients *C*(*k*) for nodes with degree=*k* in a hierarchical network follows a power-law (i.e., *C*(*k*)∝*k*^-^*^r^*, where *r*≒1) [30,31]). In the IVV network, the value of *r* was 0.82 (*R*^2^=0.52), whereas it was close to 0.81 (*R*^2^=0.54) in the Y2H network [7] (Table S9), indicating that the IVV network displays a hierarchical topology.

Neighbor connectivity is the mean degree of all neighboring nodes. The mean neighbor connectivity for nodes with degree=*k,* *N*(*k*), follows a power-law with exponent *r* (i.e*., N*(*k*)∝*k*^-^*^r^*) in large-scale PPI networks obtained from high-throughput experiments, such as Y2H [32]. In contrast to the case of a Y2H network with *r*=0.32 (*R*^2^=0.55) [8], the value of *r* in the IVV network was 0.85 (*R*^2^=0.53). Furthermore, the value of *r* in the network of LC PPIs related to the 50 TF proteins is 0.70 with *R*^2^=0.73 (Table S9). These results suggest that nodes with a high degree of connectivity (i.e., hubs) preferentially connect to nodes with a low degree in the IVV network.

The ‘small world’ network is characterized by the mean of the shortest path lengths between two nodes and the mean clustering coefficient for all nodes [7]. The mean shortest path length between two nodes in the IVV network is 4.35 (Table S9). This value is significantly smaller than the value for an artificially created random network consisting of the same number of nodes (730) and edges (943) as the IVV network, 6.7±0.2. In addition, the mean clustering coefficient of all nodes in the IVV network is 0.005, close to the value for the Y2H network, 0.006 ([8]; Table S9). The value calculated for the network consisting of LC PPIs for the same 50 TF proteins used as bait in our experiment is 0.099. Additionally, the value for the whole network of the LC PPIs is 0.105. Considering that the value obtained from the IVV network (0.005) is small compared with the previous two values (0.099 and 0.105; Table S9), the IVV network cannot be considered a small world network, a characteristic that has been previously discussed for the Y2H network [7]. The path length in the IVV network is characteristically short, but unlike small world networks of protein interactions in other organisms, it does not exhibit a high clustering coefficient (Table S9). One possible explanation for this discrepancy is the fact that we sampled a limited subset of links from the complete network. This could result in a smaller increase in the clustering coefficient than for random networks [7].

As stated above, the IVV network presented in this paper exhibited considerable similarity to the Y2H network in terms of degree distribution, scale-free character, hierarchical structure, neighbor connectivity, and the mean distance between two nodes.

# VIII. Correlation of the expression of interacting proteins obtained by IVV

Previous studies have shown that interacting proteins are likely to be under similar transcriptional control (i.e., interacting pairs are likely to show similar expression profiles) [33]. This similarity may be due to the fact that protein pairs must be simultaneously expressed in order to interact. To investigate whether interacting pairs obtained from IVV core data reflect this phenomenon, we calculated expression correlations of these pairs for human transcriptome data obtained from various tissues and cells using SymAtlas (http://wombat.gnf.org/downloads/GNF1Hdata.zip; [34]) (Figure S9). We found that interacting pairs are more likely to be co-expressed than randomly selected pairs. Although the IVV data are *in vitro* data, this result suggests that the IVV data could reflect phenomena that occur *in vivo*.

**IX. Gene classification by Gene Ontology (GO)**

In order to characterize prey sequences, we assessed the GO annotations for our data set and a Y2H-derived data set [8]. In addition, a human proteome (http://cvsweb.geneontology.org/cgi-bin/cvsweb.cgi/go/gene-associations/gene_association.goa_human.gz?rev=HEAD) data set was used as a reference for the standard distribution of GO terms. Each GO identifier in each data set was counted for three main categories of ontology, ‘Molecular function’, ‘Biological process’, and ‘Cellular component’ (Figure S10A-C). Although no marked differences were observed between our data set (class 1) and the others evaluated (Y2H-derived and human proteome), there were differences between limited data sets. For example, in the limited data set composed of genes (proteins) belonging to class 1 (Figure S10A, IVV(1)) or overlapping with Pfam domain/motifs (Figure S10A, IVV(2)), transcription-related GO terms such as ‘DNA binding’, ‘transcription factor activity’, and ‘transcription regulator activity’, were more frequently identified. Similarly, such transcription-related terms appeared frequently in a limited Y2H data set (Figure S10A, Y2H (TF)) consisting of prey proteins that interact with bait proteins with GO assignments of ‘transcription regulator activity’ or ‘transcription factor activity.’ We speculate that the frequent appearance of transcription-related GO terms may be related to the composition of the bait library, which was composed entirely of TF and TF-related proteins.

# X. Assessment of tissue-specific PPIs

In the IVV selection presented in this paper, a cDNA library constructed from mRNAs derived from human brain was used as a pool of prey. Therefore, PPIs identified in our experiment are expected to have some brain-specific features. In order to test whether this is the case, we compared the frequency of brain-specific PPIs with that of liver-, lung-, kidney-, and heart-specific PPIs. Figure S11 shows the frequencies of tissue-specific PPIs in each analytical space. This frequency is defined as the product of the number of baits and the number of tissue-specific genes. As expected, brain-specific PPIs were detected more frequently than other tissue-specific PPIs in our experiment. Fisher’s exact probability test using 2x2 contingency tables (Table S10) did not indicate any significant difference among liver-, lung-, kidney-, and heart-specific PPI groups (*p*-value>0.6). However, significant differences were noted between brain-specific PPIs and the other four groups (brain – liver: *p*-value << 0.0001, brain – lung: *p*-value << 0.0001, brain – kidney: *p*-value << 0.0001, brain – heart: *p*-value << 0.0001).

# XI. Analyses of ordered/disordered regions at TF complex interfaces

Since Fischer’s lock-and-key proposal, many proteins have been identified as counterexamples for which three-dimensional structure must not be present for proper functionality. We can no longer ignore the role of a lack of three-dimensional structure (related to disordered regions) in protein-protein interaction. We suggest that the protein-structure-function paradigm [35,36,37] should be reassessed. Because we obtained interaction information as IR data at the amino acid level, we can analyze the frequency of ordered/disordered regions [38] using DISOPRED2 [39] to facilitate understanding of the protein interactions involved in TF complexes in detail (Figure 4 and Table S11). We found that the rate of disordered regions in known TF PPI data (Figure 4, LC (50 TFs)) was higher than that for all human proteins (Figure 4, All Refseq). This finding is reasonable because an association between TF function and intrinsic disorder has been reported [35]. Furthermore, similar rates of disordered regions were found for IVV PPI data (Figure 4, IVV core) and known TF PPI data (Figure 4, LC (50 TFs)), suggesting that the IVV data do not suffer from experimental bias. It is notable that the IVV IR data indicated a clearly higher rate of disordered regions than did the IVV PPI data (Figure 4, IVV core (IR)). This finding suggests that disordered regions might be more frequently involved at interfaces of TF complexes. This suggestion is consistent with the idea that disordered regions might enable TF proteins to partner with many other proteins and ligands, such as DNA [35,40,41,42]. Based on these findings, we believe that human TF complexes frequently employ disordered regions, especially at the interface level, to achieve the necessary dynamics and diversity for protein interaction.

Until recently, the lack of success in identifying small molecules that disrupt protein-protein interactions led to several misconceptions about prospects for the discovery of new drugs [43]. One myth is that the large and flat contact surfaces seen in protein complexes are rigid and do not present cavities for small molecules to bind [43]. However, in the case of interaction interfaces that have some adaptability and flexibility, it is possible that cavities exist in which small molecules may bind. A second common misconception is that native protein complexes have a higher affinity than protein-small molecule complexes and, therefore, their formation cannot be blocked by competition. However, when interaction interfaces have an inherently wobbly character, small molecules can penetrate the dynamic encounter region due to their kinetic advantage over a large therapeutic agent such as an antibody. Thus, a better understanding of interfaces involved in interaction and complex formation at the IR level (amino acid level) is needed to ensure that network analysis and biological understanding, including intrinsic disorder properties, are connected [10]. This line of thought could contribute to the discovery of flexible interfaces with hot spots [44] that might be suitable for the rational design of new therapeutic agents [43].

**Supporting Methods**

## Modified preparation of bait mRNA templates

We prepared 68 bait proteins representing 50 human TF proteins (Table S1). All 68 cDNA fragments (full length and/or partial domains of the TF proteins) were amplified by a four-step PCR with exTaq (Takara Bio) using a Qiagen Biorobot 8000. The PCR was performed as shown in Figure S1 and Table S1-3. In the first PCR step, cDNA fragments were amplified with a forward primer (primer 1) that included a T7 tag and a reverse primer (primer 2) that contained part of the affinity tag (Table S2). In the second PCR step, preparation of the affinity tag fragment was performed by PCR with 5'baitCBP (primer 3) and 3'FosCBPzz (primer 4) primers under the conditions of program No. 1 (Table S3). In the third PCR step, the overlap PCR was carried out with the affinity tag primers and cDNA fragments containing part of the affinity tag. In the fourth PCR step, the PCR products obtained from the third step were connected to the promoter region with primer 5 so that they could be used as bait cDNA templates. Finally, the fourth step PCR products were transcribed into RNA. The PCR products were purified with QIAquick 96 Plates (Qiagen) and a QIAquick PCR Purification Kit (Qiagen)[11]. The mRNA templates were prepared with a RiboMAX Large Scale RNA Production System-SP6 (Promega) and m7G(5')ppp(5')G RNA Capping Analog (Invitrogen Corp., Carlsbad, CA, USA)[11] and then purified with an RNeasy96BioRobot8000Kit (Qiagen) and an RNeasy Mini Kit (Qiagen)[11]. The mRNAs were detected by routine western blot analysis using the anti-T7 antibody. Ninety-six percent of bait proteins were expressed in the *in vitro* translation system using this method (Table S1).

***In vitro* parallel auto-selection using IVV**

A human brain cDNA library was prepared to be used as prey in parallel auto-selection using 96-well plates, carried out using the Qiagen Biorobot 8000 according to a previously described method [11]. We purchased commercially available human brain mRNA from the BioChain Institute, Inc. According to the reported method [19], large-scale synthesis of a PEG Puro spacer was performed by Invitrogen Japan K.K., Tokyo, Japan and Takara Bio Inc., Otsu, Japan. We prepared 68 bait mRNA templates for 50 human TF proteins as described in the “Preparation of bait mRNA templates” section. A human brain cDNA library was used as prey, and the 68 mRNA templates used as bait were co-translated for 1 h at 26˚C in 96-well plates using a Qiagen Biorobot 8000 and a wheat germ extract translation system (Zoegene Corporation, now Molecuence Corporation), with 200 nM RNA template for prey and 400 nM mRNA template for bait. After each round of selection, RT-PCR was performed. The RT-PCR products were purified using QIAquick 96 Plates (Qiagen) and then used as templates for an *in vitro* transcription reaction performed with a RiboMAX Large Scale RNA Production System-SP6 (Promega) and m7G(5')ppp(5')G RNA Capping Analog (Invitrogen Corp., Carlsbad, CA, USA). To prepare the prey RNA templates of IVV for the next round of selection, the purified mRNA products obtained from an RNeasy96BioRobot8000Kit (Qiagen) were ligated to the PEG Puro spacer [19] in 96-well plates. After six rounds of selection, the obtained sequences were identified by Takara　Bio　Inc.,　Otsu,　Japan and Shimadzu Corporation, Kyoto, Japan. A mock experiment without bait protein was run as a negative control to eliminate technical false positives in the IST analysis. To monitor enrichment during selection, we carried out a control experiment using full-length ETS1 as bait. Because JUN is known to interact with ETS1, we were able to follow the enrichment of JUN using PCR to monitor prey DNA templates after each round of selection. 5'preyJun (CCGAAAAAGGAAGCTGGAGAGAA) and 3'preyJun (ACTTTCTGTTTAAGCTGTGCCACC) primers were used to amplify Jun DNA. Amplification conditions were as follows: 35 cycles of denaturation at 98°C for 30 s, primer annealing at 62°C for 30 s, and primer extension at 73°C for 30 s. The PCR products were subjected to electrophoresis on a 2% agarose gel. The process of high-throughput parallel 96-well selection takes approximately one month, which is equivalent to performing over 3 selections per day.

## Analyses of network characteristics

Degree distributions were analyzed in order to estimate whether the PPI network displays scale-free characteristics, as has been performed in other studies [30,31]. In order to assess the hierarchical property of the IVV core data network, we used the clustering coefficient, *C*. The coefficient *C* of a node *p* with degree=*k* was expressed as the number of closed triangle loops containing *p* divided by the number of combinations (i.e., *C_p_=2n/k* (*k* – 1)). This analytical method was described in previous reports [7,31]. Neighbor connectivity was calculated as the mean degree of neighboring nodes for each node [32]. The mean distance between two nodes was calculated as the mean shortest path length between all possible pairs. The literature-curated (LC) human PPI data set was downloaded from the NCBI ftp site (ftp://ftp.ncbi.nlm.nih.gov/gene/GeneRIF/interactions.gz, as of July 27, 2006).

## Analysis of expression correlations among the IVV core data

The core data set was obtained from the overall IVV PPI data, which is generated from a set of prey and bait proteins. For each pair of prey and bait proteins, we identified the corresponding pair of NCBI gene IDs. We then obtained the expression patterns of the gene pairs from SymAtlas [34], a collection of expression patterns of human and mouse genes in various tissues. The expression data “Human U133A + GNF1H (MAS5-condensed)” were used for the analysis. Finally, we calculated Pearson’s correlation coefficients for all gene pairs and generated a histogram of these coefficients. In the case of multiple probes for a single gene, we normalized the expression patterns for each probe by calculating Z-scores. The normalized expression patterns were then averaged and treated as the expression pattern for the gene.

## Analyses of tissue-specific PPIs

A data set of tissue-specific genes was created based on the gene expression data from SymAtlas (http://wombat.gnf.org/downloads/GNF1Hdata.zip, as of March 5, 2005). We initially selected probes from the dataset with intensity values equal to or greater than 500. Five tissues were analyzed: whole brain, lung, liver, kidney, and heart. We subsequently selected the genes that corresponded to the selected probes. Second, genes that appeared in two or more tissues were excluded, and the remaining genes were defined as tissue-specific. Finally, we counted PPIs involving one or two tissue-specific genes (proteins) as tissue-specific PPIs.

# Supporting References

1. Uetz P, Giot L, Cagney G, Mansfield TA, Judson RS, et al. (2000) A comprehensive analysis of protein-protein interactions in Saccharomyces cerevisiae. Nature 403: 623-627.

2. Ito T, Chiba T, Ozawa R, Yoshida M, Hattori M, et al. (2001) A comprehensive two-hybrid analysis to explore the yeast protein interactome. Proc Natl Acad Sci U S A 98: 4569-4574.

3. Gavin AC, Bosche M, Krause R, Grandi P, Marzioch M, et al. (2002) Functional organization of the yeast proteome by systematic analysis of protein complexes. Nature 415: 141-147.

4. Giot L, Bader JS, Brouwer C, Chaudhuri A, Kuang B, et al. (2003) A protein interaction map of Drosophila melanogaster. Science 302: 1727-1736.

5. Li S, Armstrong CM, Bertin N, Ge H, Milstein S, et al. (2004) A map of the interactome network of the metazoan C. elegans. Science 303: 540-543.

6. Butland G, Peregrin-Alvarez JM, Li J, Yang W, Yang X, et al. (2005) Interaction network containing conserved and essential protein complexes in Escherichia coli. Nature 433: 531-537.

7. Rual JF, Venkatesan K, Hao T, Hirozane-Kishikawa T, Dricot A, et al. (2005) Towards a proteome-scale map of the human protein-protein interaction network. Nature 437: 1173-1178.

8. Stelzl U, Worm U, Lalowski M, Haenig C, Brembeck FH, et al. (2005) A human protein-protein interaction network: a resource for annotating the proteome. Cell 122: 957-968.

9. Han JD, Bertin N, Hao T, Goldberg DS, Berriz GF, et al. (2004) Evidence for dynamically organized modularity in the yeast protein-protein interaction network. Nature 430: 88-93.

10. Hakes L, Pinney JW, Robertson DL, Lovell SC (2008) Protein-protein interaction networks and biology--what's the connection? Nat Biotechnol 26: 69-72.

11. Miyamoto-Sato E, Ishizaka M, Horisawa K, Tateyama S, Takashima H, et al. (2005) Cell-free cotranslation and selection using in vitro virus for high-throughput analysis of protein-protein interactions and complexes. Genome Res 15: 710-717.

12. Fields S, Song O (1989) A novel genetic system to detect protein-protein interactions. Nature 340: 245-246.

13. Rigaut G, Shevchenko A, Rutz B, Wilm M, Mann M, et al. (1999) A generic protein purification method for protein complex characterization and proteome exploration. Nat Biotechnol 17: 1030-1032.

14. Huang C, Morcos F, Kanaan SP, Wuchty S, Chen DZ, et al. (2007) Predicting protein-protein interactions from protein domains using a set cover approach. IEEE/ACM Trans Comput Biol Bioinform 4: 78-87.

15. Miyamoto-Sato E, Nemoto N, Kobayashi K, Yanagawa H (2000) Specific bonding of puromycin to full-length protein at the C-terminus. Nucleic Acids Res 28: 1176-1182.

16. Nemoto N, Miyamoto-Sato E, Husimi Y, Yanagawa H (1997) In vitro virus: bonding of mRNA bearing puromycin at the 3'-terminal end to the C-terminal end of its encoded protein on the ribosome in vitro. FEBS Lett 414: 405-408.

17. Roberts RW, Szostak JW (1997) RNA-peptide fusions for the in vitro selection of peptides and proteins. Proc Natl Acad Sci U S A 94: 12297-12302.

18. Hammond PW, Alpin J, Rise CE, Wright M, Kreider BL (2001) In vitro selection and characterization of Bcl-X(L)-binding proteins from a mix of tissue-specific mRNA display libraries. J Biol Chem 276: 20898-20906.

19. Miyamoto-Sato E, Takashima H, Fuse S, Sue K, Ishizaka M, et al. (2003) Highly stable and efficient mRNA templates for mRNA-protein fusions and C-terminally labeled proteins. Nucleic Acids Res 31: e78.

20. Horisawa K, Tateyama S, Ishizaka M, Matsumura N, Takashima H, et al. (2004) In vitro selection of Jun-associated proteins using mRNA display. Nucleic Acids Res 32: e169.

21. Shen X, Valencia CA, Szostak JW, Dong B, Liu R (2005) Scanning the human proteome for calmodulin-binding proteins. Proc Natl Acad Sci U S A 102: 5969-5974.

22. Tong AH, Drees B, Nardelli G, Bader GD, Brannetti B, et al. (2002) A combined experimental and computational strategy to define protein interaction networks for peptide recognition modules. Science 295: 321-324.

23. Ewing RM, Chu P, Elisma F, Li H, Taylor P, et al. (2007) Large-scale mapping of human protein-protein interactions by mass spectrometry. Mol Syst Biol 3: 89.

24. Walhout AJ, Vidal M (1999) A genetic strategy to eliminate self-activator baits prior to high-throughput yeast two-hybrid screens. Genome Res 9: 1128-1134.

25. Albers M, Kranz H, Kober I, Kaiser C, Klink M, et al. (2005) Automated yeast two-hybrid screening for nuclear receptor-interacting proteins. Mol Cell Proteomics 4: 205-213.

26. Finn RD, Mistry J, Schuster-Bockler B, Griffiths-Jones S, Hollich V, et al. (2006) Pfam: clans, web tools and services. Nucleic Acids Res 34: D247-251.

27. Chinenov Y, Kerppola TK (2001) Close encounters of many kinds: Fos-Jun interactions that mediate transcription regulatory specificity. Oncogene 20: 2438-2452.

28. Chen L, Glover JN, Hogan PG, Rao A, Harrison SC (1998) Structure of the DNA-binding domains from NFAT, Fos and Jun bound specifically to DNA. Nature 392: 42-48.

29. Legrain P, Selig L (2000) Genome-wide protein interaction maps using two-hybrid systems. FEBS Lett 480: 32-36.

30. Ravasz E, Somera AL, Mongru DA, Oltvai ZN, Barabasi AL (2002) Hierarchical organization of modularity in metabolic networks. Science 297: 1551-1555.

31. Barabasi AL, Oltvai ZN (2004) Network biology: understanding the cell's functional organization. Nat Rev Genet 5: 101-113.

32. Maslov S, Sneppen K (2002) Specificity and stability in topology of protein networks. Science 296: 910-913.

33. Ge H, Liu Z, Church GM, Vidal M (2001) Correlation between transcriptome and interactome mapping data from Saccharomyces cerevisiae. Nat Genet 29: 482-486.

34. Su AI, Cooke MP, Ching KA, Hakak Y, Walker JR, et al. (2002) Large-scale analysis of the human and mouse transcriptomes. Proc Natl Acad Sci U S A 99: 4465-4470.

35. Dunker AK, Obradovic Z (2001) The protein trinity--linking function and disorder. Nat Biotechnol 19: 805-806.

36. Dyson HJ, Wright PE (2005) Intrinsically unstructured proteins and their functions. Nat Rev Mol Cell Biol 6: 197-208.

37. Radivojac P, Iakoucheva LM, Oldfield CJ, Obradovic Z, Uversky VN, et al. (2007) Intrinsic disorder and functional proteomics. Biophys J 92: 1439-1456.

38. Oldfield CJ, Cheng Y, Cortese MS, Romero P, Uversky VN, et al. (2005) Coupled folding and binding with alpha-helix-forming molecular recognition elements. Biochemistry 44: 12454-12470.

39. Ward JJ, Sodhi JS, McGuffin LJ, Buxton BF, Jones DT (2004) Prediction and functional analysis of native disorder in proteins from the three kingdoms of life. J Mol Biol 337: 635-645.

40. Patil A, Nakamura H (2006) Disordered domains and high surface charge confer hubs with the ability to interact with multiple proteins in interaction networks. FEBS Lett 580: 2041-2045.

41. Haynes C, Oldfield CJ, Ji F, Klitgord N, Cusick ME, et al. (2006) Intrinsic disorder is a common feature of hub proteins from four eukaryotic interactomes. PLoS Comput Biol 2: e100.

42. Singh GP, Ganapathi M, Dash D (2007) Role of intrinsic disorder in transient interactions of hub proteins. Proteins 66: 761-765.

43. Wells JA, McClendon CL (2007) Reaching for high-hanging fruit in drug discovery at protein-protein interfaces. Nature 450: 1001-1009.

44. Moreira IS, Fernandes PA, Ramos MJ (2007) Hot spots--a review of the protein-protein interface determinant amino-acid residues. Proteins 68: 803-812.

45. Shannon P, Markiel A, Ozier O, Baliga NS, Wang JT, et al. (2003) Cytoscape: a software environment for integrated models of biomolecular interaction networks. Genome Res 13: 2498-2504.
