# Supplementary figures and images for: A Comprehensive Resource of Interacting Protein Regions for Refining Human Transcription Factor Networks
Source: PLoS One. 2010 Feb 24;5(2):e9289. doi: 10.1371/journal.pone.0009289 (PMC2827538; doi:10.1371/journal.pone.0009289)

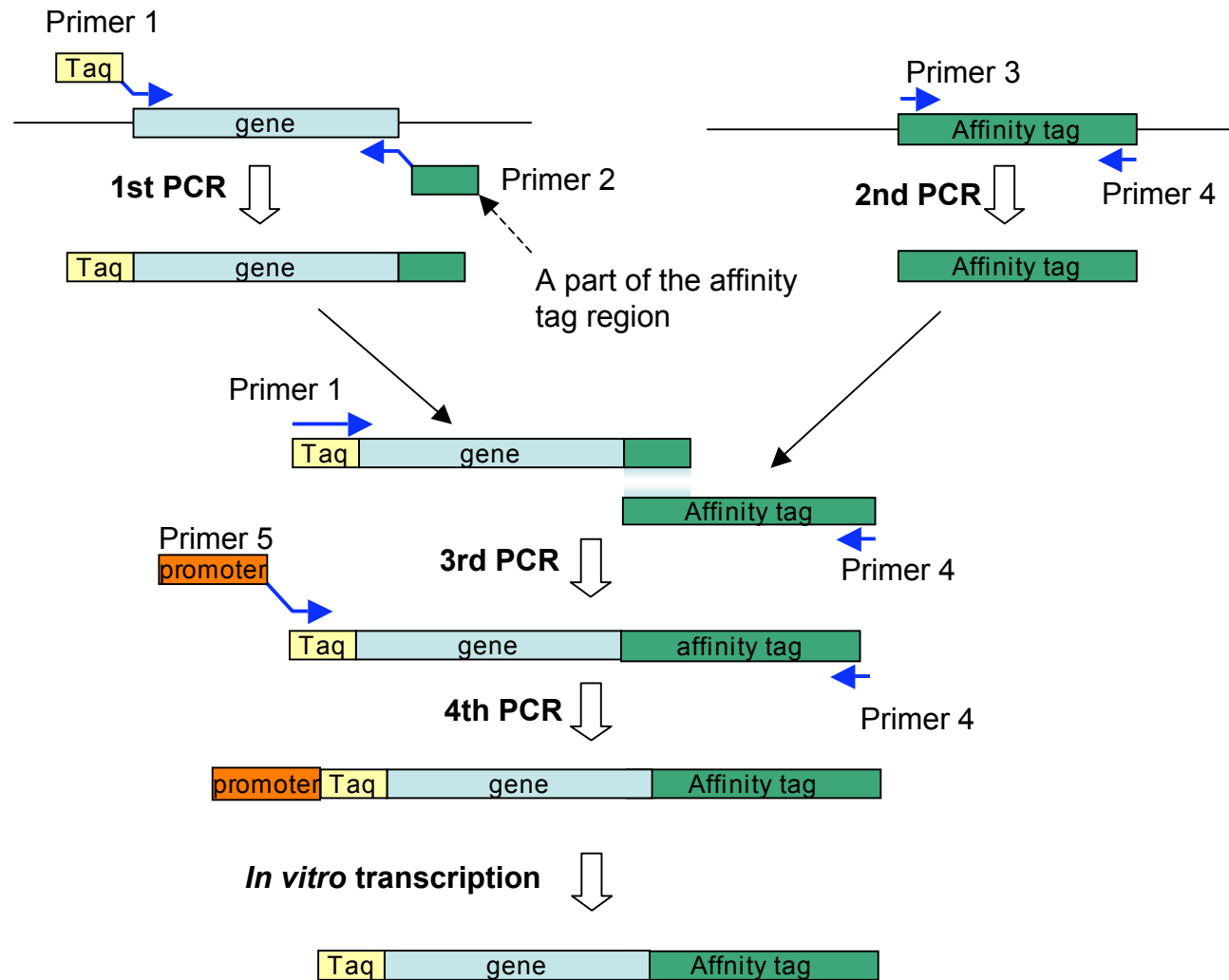

Supplement: Figure S1 — Strategy of mRNA preparation for bait proteins. Primer 1 consists of a gene-specific sequence (sky-blue box) and the T7 tag (yellow box) sequence. Primer 2 consists of a gene-specific sequence and part of the affinity tag sequence (green box). These primers were used to connect a gene with tag sequences. The tagged construct was amplified by primers 3 and 4. Primer 5 consists of the promoter (orange box) and the T7 tag sequence. Primers 4 and 5 were used to connect a gene to a promoter. A bait protein encoding mRNA was then transcribed from the 4th PCR product. (0.04 MB PDF) [file pone.0009289.s002.pdf]

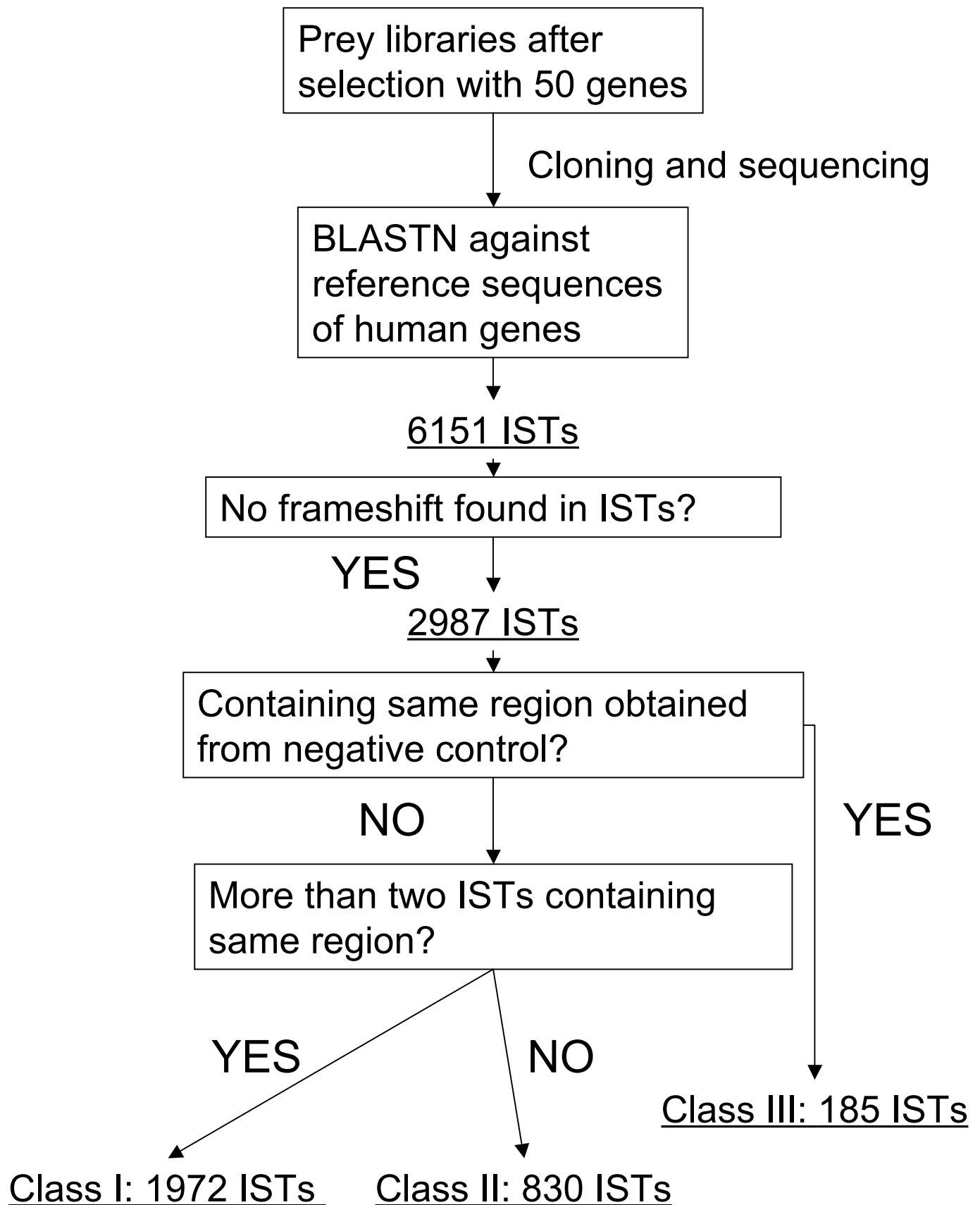

Supplement: Figure S2 — Flow chart of interacting sequence tag (IST) analysis after IVV selection. ISTs of prey proteins were detected, evaluated for quality of alignment to reference sequences of human genes and subdivided into 3 classes. See ‘IST analysis’ in the Supporting Methods section. (0.03 MB PDF) [file pone.0009289.s003.pdf]

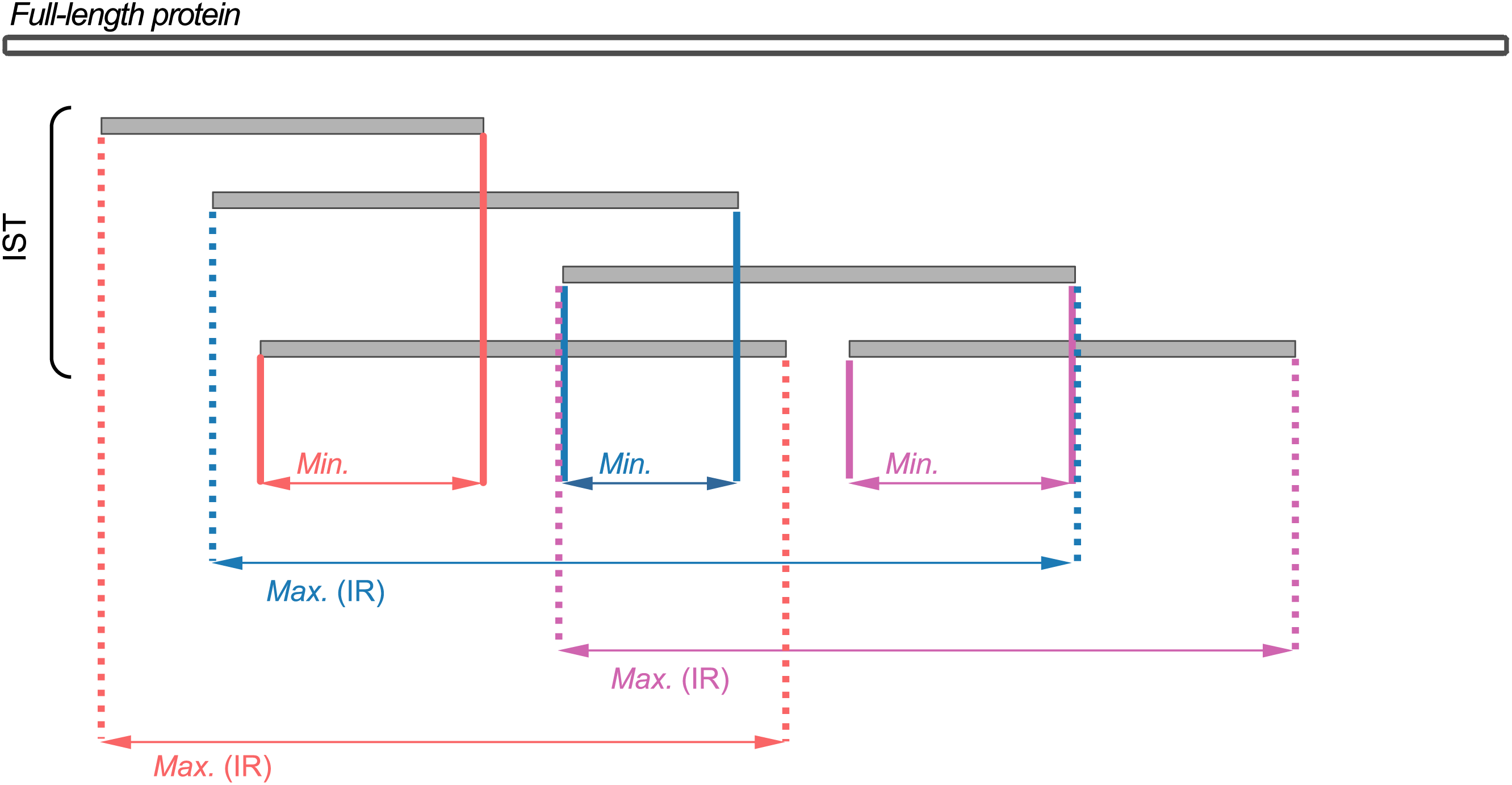

Supplement: Figure S3 — Definition of interacting regions (IR) and clusters. An example of interacting regions (IRs) determined by five different ISTs (indicated by the shaded boxes) is shown. In the presented case, there are three clusters containing the minimum/maximum regions for each IR. The maximum regions correspond to IRs containing interacting domains and/or motifs. Colors (red, blue, purple) correspond to each cluster. (0.33 MB TIF) [file pone.0009289.s004.tif]

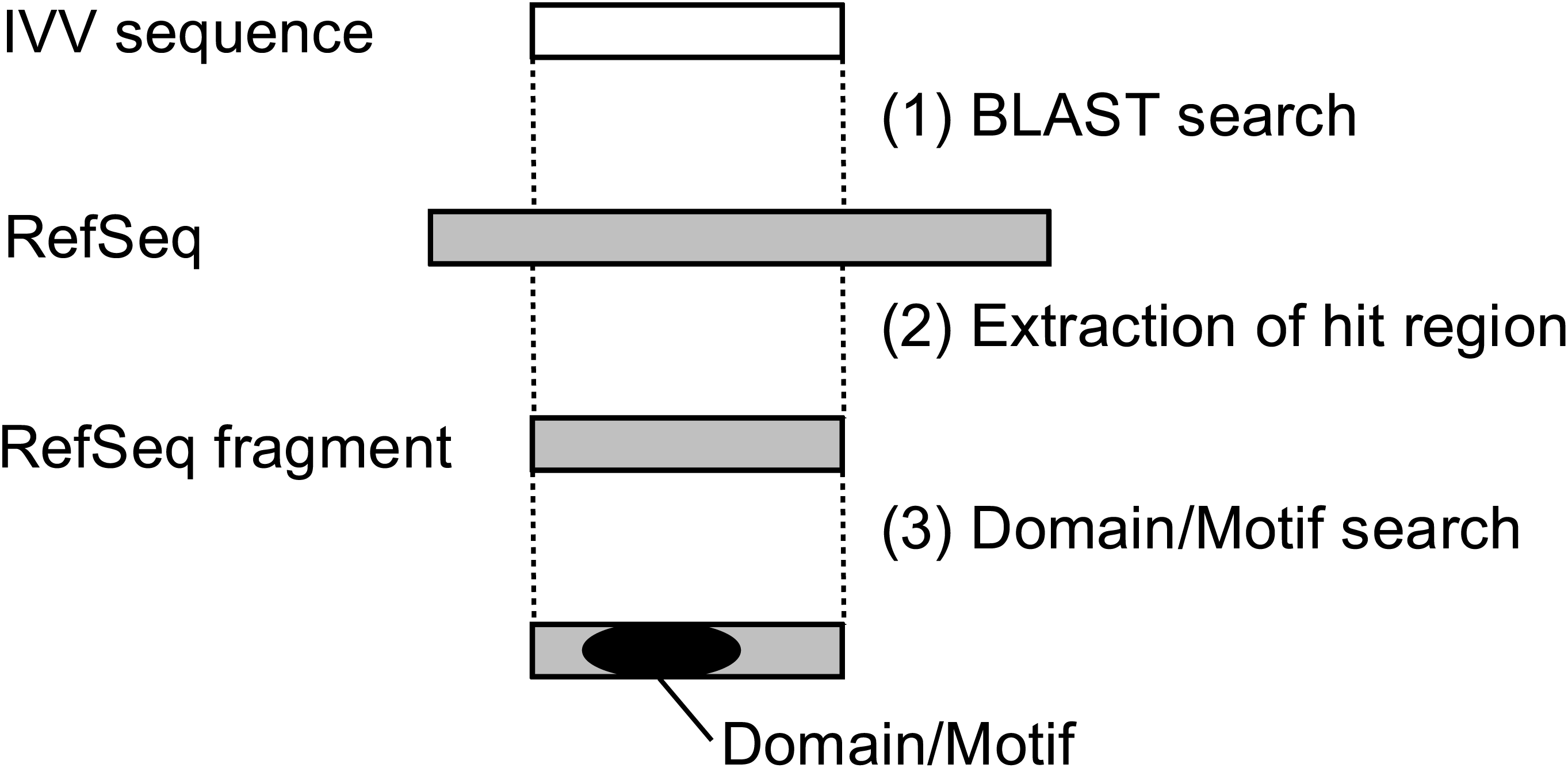

Supplement: Figure S5 — Procedure for identification of known protein domains/motifs in IVV IRs by a Pfam search with “hmmpfam.” Motifs were identified using the following 3 steps: (1) A BLAST search of each IVV sequence against the human RefSeq protein database; (2) Extraction of the RefSeq protein fragment corresponding to a hit region; and (3) Searching for the motif(s) in each protein fragment. The “hmmpfam” was used to find known protein motifs in the Pfam database. (0.15 MB TIF) [file pone.0009289.s006.tif]

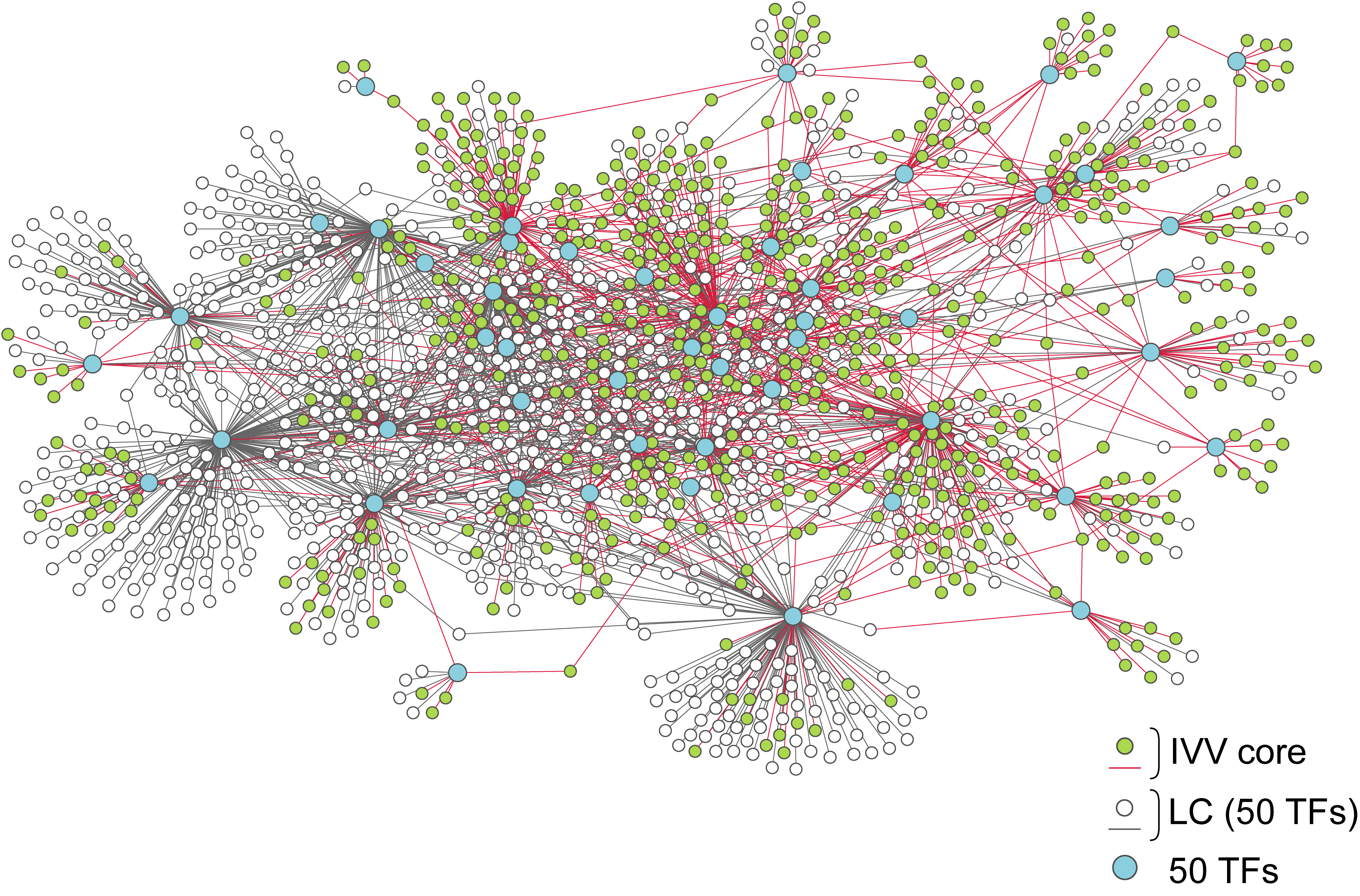

Supplement: Figure S7 — PPI network focused on 50 human TF proteins. A merged network of IVV core data and LC PPI data focused on 50 human TFs. Nodes corresponding to the 50 TFs are indicated in blue. LC PPIs are indicated by black edges and white nodes in the graph. Newly identified PPIs are indicated by red edges and green nodes (see Data VI). (2.61 MB TIF) [file pone.0009289.s008.tif]

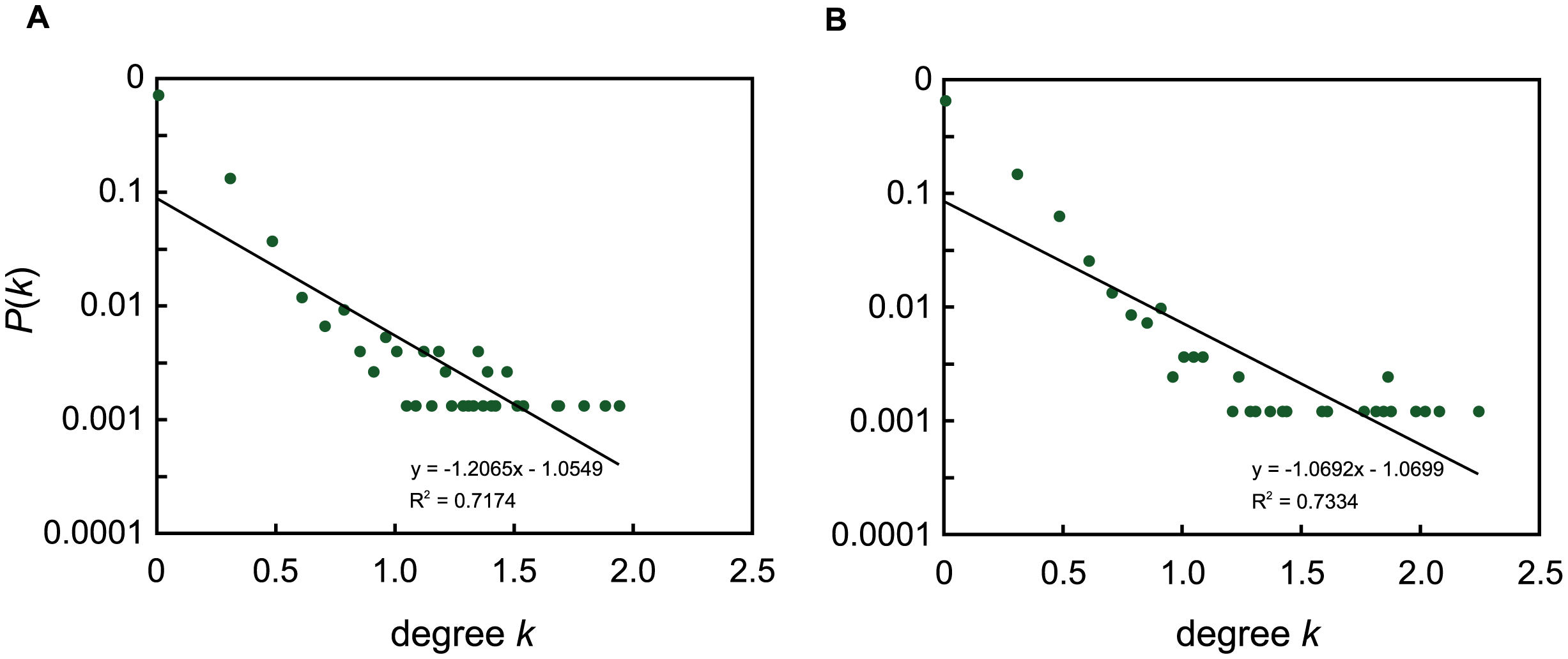

Supplement: Figure S8 — Degree distributions. (A) Degree distribution of the nodes in the PPI network generated from IVV data; (B) Degree distribution of the nodes in the network generated from LC data on PPIs directly related to the 50 TF proteins used as bait in the IVV experiments. (0.27 MB TIF) [file pone.0009289.s009.tif]

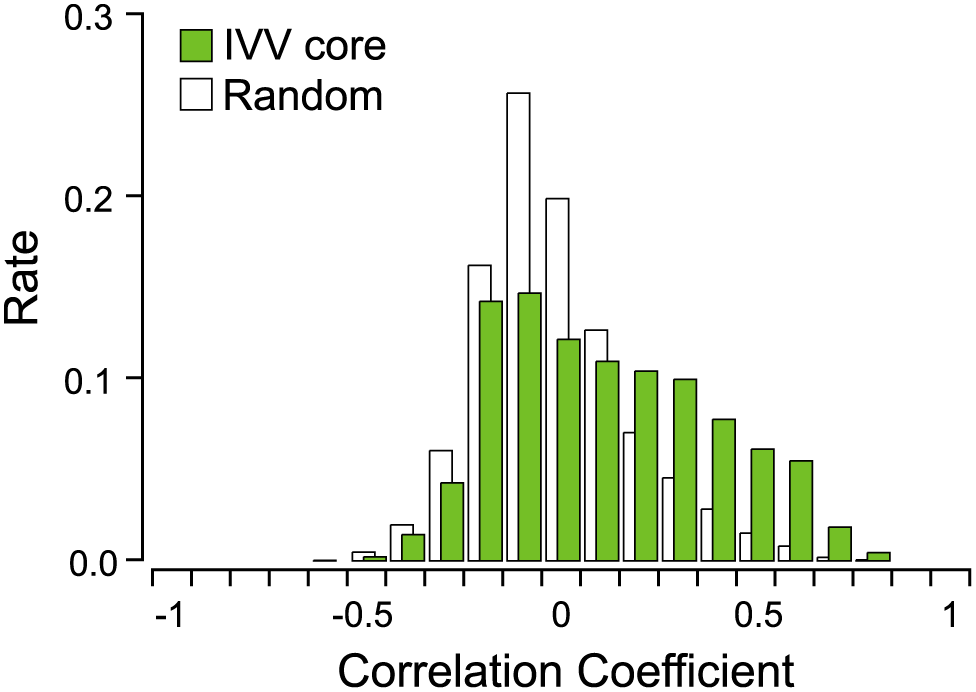

Supplement: Figure S9 — Expression correlations of PPIs obtained with the IVV method. The horizontal and vertical axes show expression correlations of interacting pairs and their rates among all of the pairs, respectively. (0.13 MB TIF) [file pone.0009289.s010.tif]

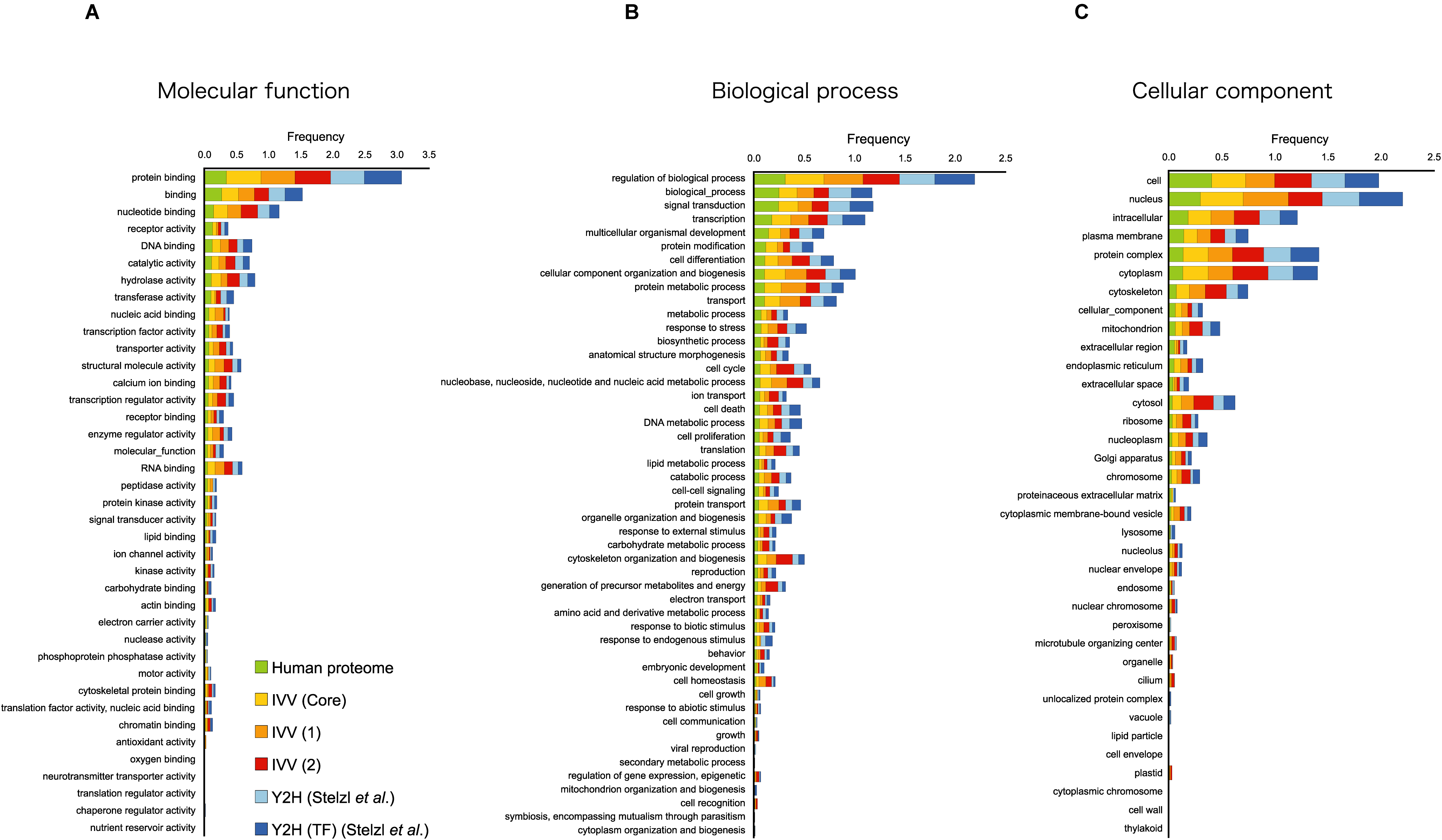

Supplement: Figure S10 — Gene classification by Gene Ontology (GO). The frequencies of the GO terms from the following five data sets are shown: (1) Human proteome (http://cvsweb.geneontology.org/cgi-bin/cvsweb.cgi/go/gene-associations/gene_association.goa_human.gz?rev=HEAD), IVV (Core), (2) the dataset limited to prey genes (proteins) belonging to class 1; (3) the dataset limited to prey genes (proteins) having any motif/domain in the IST regions; (4) the Y2H data set including genes (proteins) obtained as the prey; and (5) the Y2H; TF) data set limited to the prey genes (proteins) that interact with baits having GO assignments of ‘transcription regulator activity’ or ‘transcription factor activity.’ GO identifiers for genes in each data set were counted in three main categories of ontology: A, ‘Molecular function;’ B, ‘Biological process;’ and C, ‘Cellular component.’ GO slim files (http://www.geneontology.org/GO_slims/goslim_generic.obo) were used to summarize annotations for each data set. (1.22 MB TIF) [file pone.0009289.s011.tif]

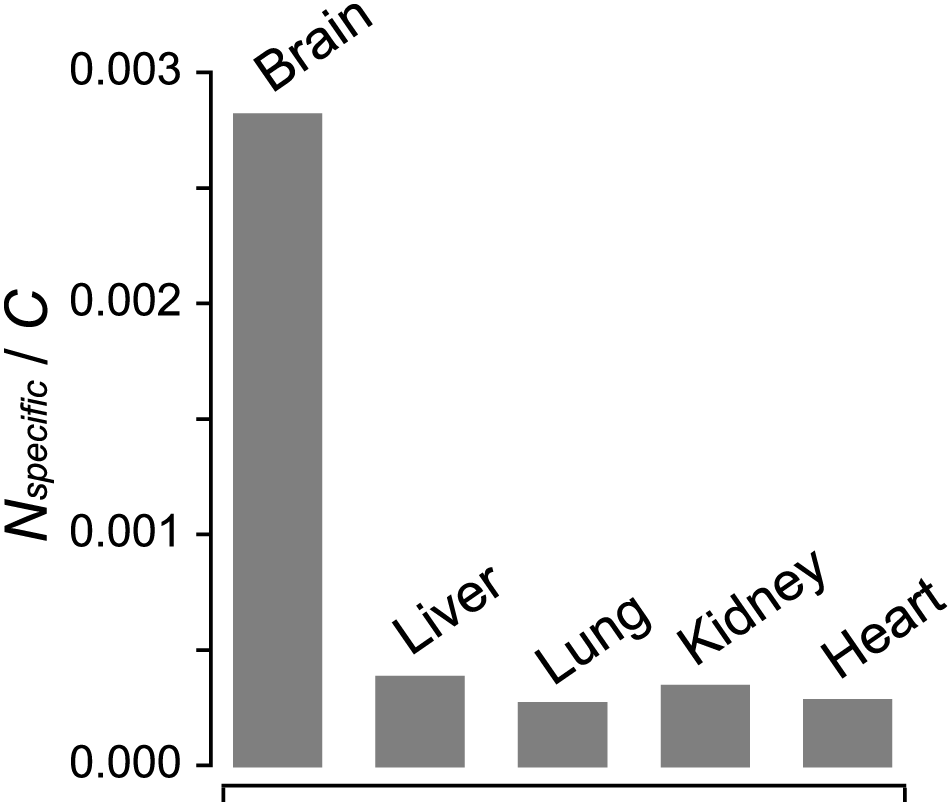

Supplement: Figure S11 — Tissue-specific PPIs. The histogram shows the proportions of tissue-specific PPIs (Data X) in the possible analytical space C, which is defined as the product of the number of tissue-specific genes and the number of bait proteins: 45,200 (904×50) in brain tissue; 25,300 (506×50) in liver tissue; 24,800 (496×50) in lung tissue; 14,050 (281×50) in kidney tissue; and 13,550 (271×50) in heart tissue. The numbers of brain-, liver-, lung-, kidney, and heart-specific PPIs, Nspecific, were 128, 10, 7, 5, and 4, respectively. (0.05 MB TIF) [file pone.0009289.s012.tif]

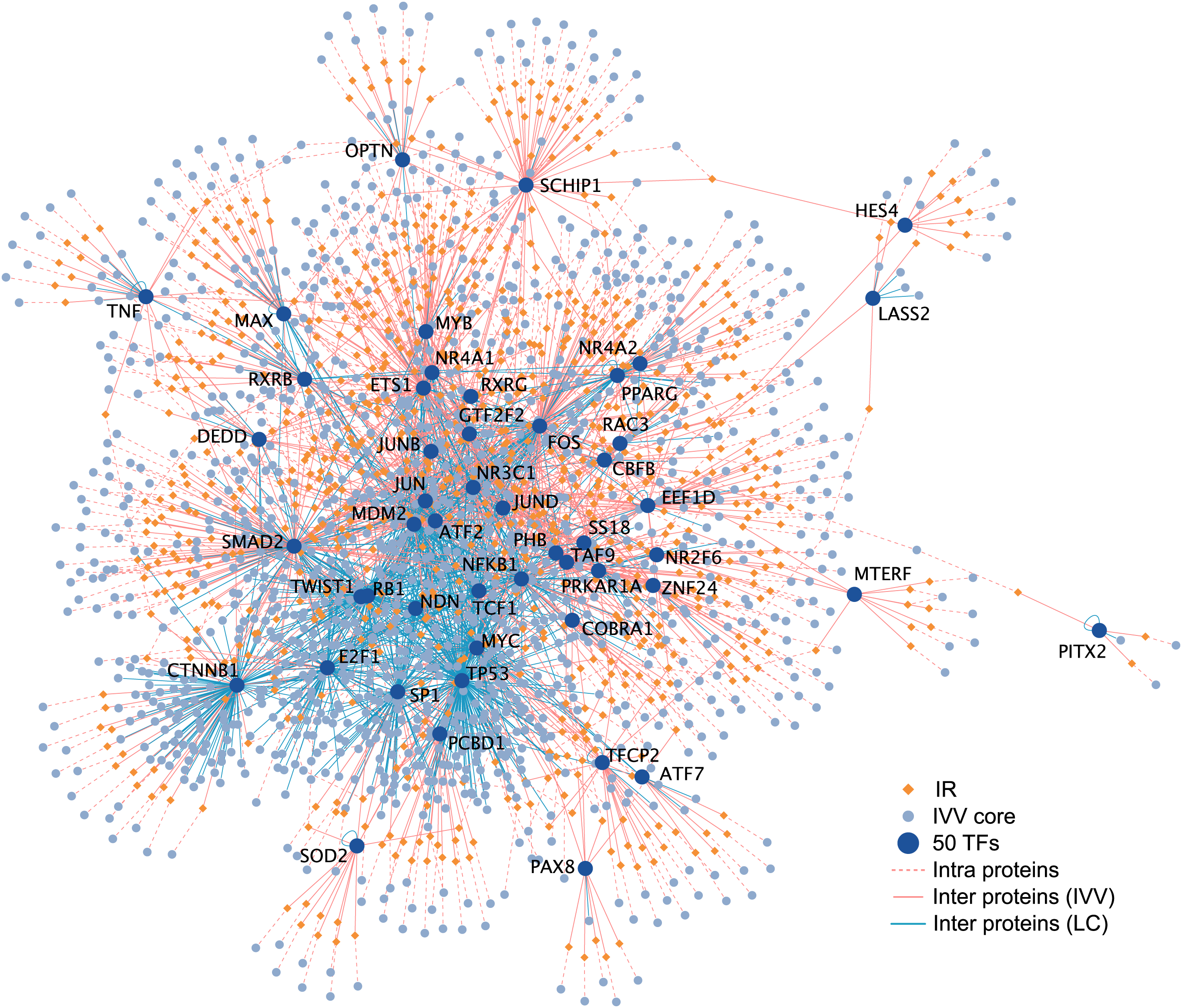

Supplement: Figure S12 — IR-level PPI network of 50 human TF proteins. A merged network of the IR-level PPI network of the IVV core data set and the LC PPI data set (1,240 LC PPIs) focused on 50 human TFs. Nodes corresponding to the 50 TFs are indicated in blue. Interactions from the IVV and LC data sets are indicated by red and blue edges in the graph, respectively (see Figure 3A). All of the network graphs were produced in Cytoscape. Cytoscape files (IVV_IR_Networks.cys'), including this figure and Figure 3A, are available upon request (contact EM-S[nekoneko@educ.cc.keio.ac.jp]). (2.82 MB TIF) [file pone.0009289.s013.tif]
