# Supplementary material for: A Comprehensive Resource of Interacting Protein Regions for Refining Human Transcription Factor Networks
Source: PLoS One. 2010 Feb 24;5(2):e9289. doi: 10.1371/journal.pone.0009289 (PMC2827538; doi:10.1371/journal.pone.0009289)
Supplement: Figure S4 — Verification of IVV PPIs (IRs) by pull-down and real-time PCR assays. (A) Results of the in vitro pull-down assay. Each pull-down assay number corresponds to a number in Table S5. Prey protein prior to elution (INPUT) and the eluate in the presence (+) and absence (−) of the bait protein are shown. (B) Real-time PCR results. The numbers correspond to the verification numbers (Table S5). The x-axis value indicates the round of selection and the y-axis value indicates the measured DNA copy number. Blue and red colors indicate the selection results with and without bait protein, respectively. (0.60 MB PDF) [file pone.0009289.s005.pdf]

A

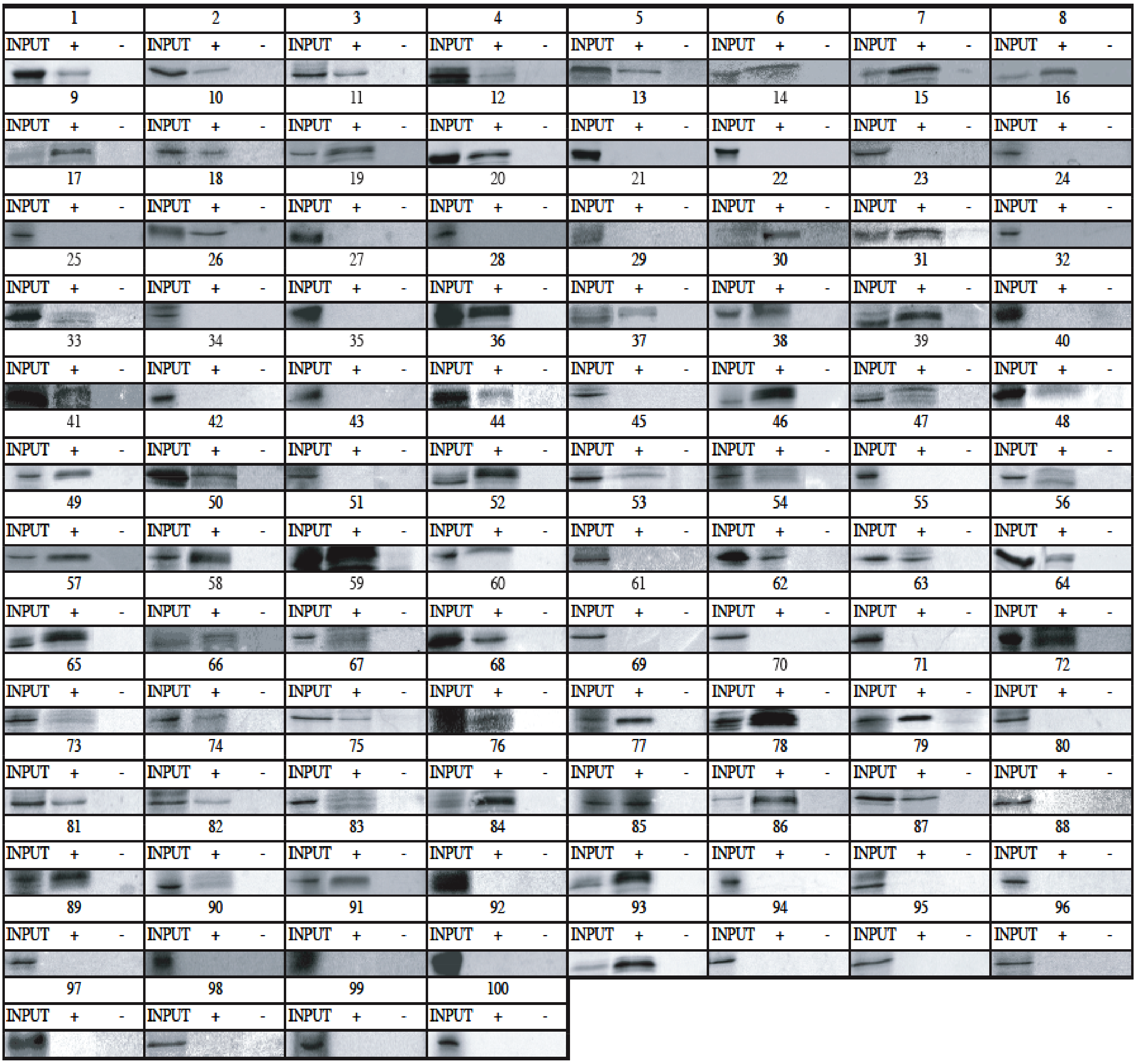
1
2
3
4
5
6
7
8
9
10
11
12
13
14
15
16
17
18
19
20
21
22
23
24
25
26
27
28
29
30
31
32
33
34
35
36
37
38
39
40
41
42
43
44
45
46
47
48
49
50
51
52
53
54
55
56
57
58
59
60
61
62
63
64
65
66
67
68
69
70
71
72
73
74
75
76
77
78
79
80
81
82
83
84
85
86
87
88
89
90
91
92
93
94
95
96
97
98
99
100
INPUT
+
-

B

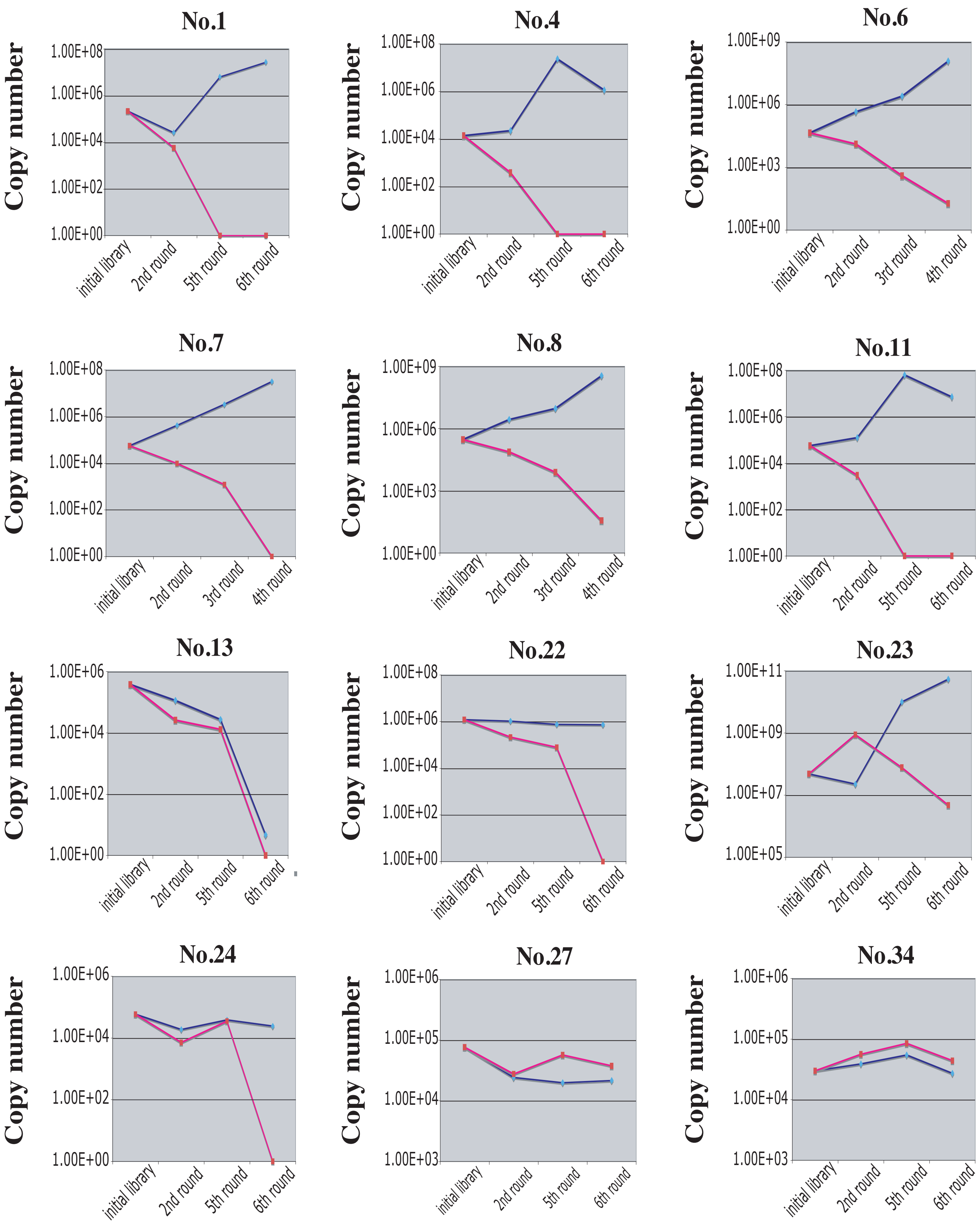
No.1
Copy number
1.00E+08
1.00E+06
1.00E+04
1.00E+02
1.00E+00
initial library
2nd round
5th round
6th round
No.4
Copy number
1.00E+08
1.00E+06
1.00E+04
1.00E+02
1.00E+00
initial library
2nd round
5th round
6th round
No.6
Copy number
1.00E+09
1.00E+06
1.00E+03
1.00E+00
initial library
2nd round
3rd round
4th round
No.7
Copy number
1.00E+08
1.00E+06
1.00E+04
1.00E+02
1.00E+00
initial library
2nd round
3rd round
4th round
No.8
Copy number
1.00E+09
1.00E+06
1.00E+03
1.00E+00
initial library
2nd round
3rd round
4th round
No.11
Copy number
1.00E+08
1.00E+06
1.00E+04
1.00E+02
1.00E+00
initial library
2nd round
5th round
6th round
No.13
Copy number
1.00E+06
1.00E+04
1.00E+02
1.00E+00
initial library
2nd round
5th round
6th round
No.22
Copy number
1.00E+08
1.00E+06
1.00E+04
1.00E+02
1.00E+00
initial library
2nd round
5th round
6th round
No.23
Copy number
1.00E+11
1.00E+09
1.00E+07
1.00E+05
initial library
2nd round
5th round
6th round
No.24
Copy number
1.00E+06
1.00E+04
1.00E+02
1.00E+00
initial library
2nd round
5th round
6th round
No.27
Copy number
1.00E+06
1.00E+05
1.00E+04
1.00E+03
initial library
2nd round
5th round
6th round
No.34
Copy number
1.00E+06
1.00E+05
1.00E+04
1.00E+03
initial library
2nd round
5th round
6th round

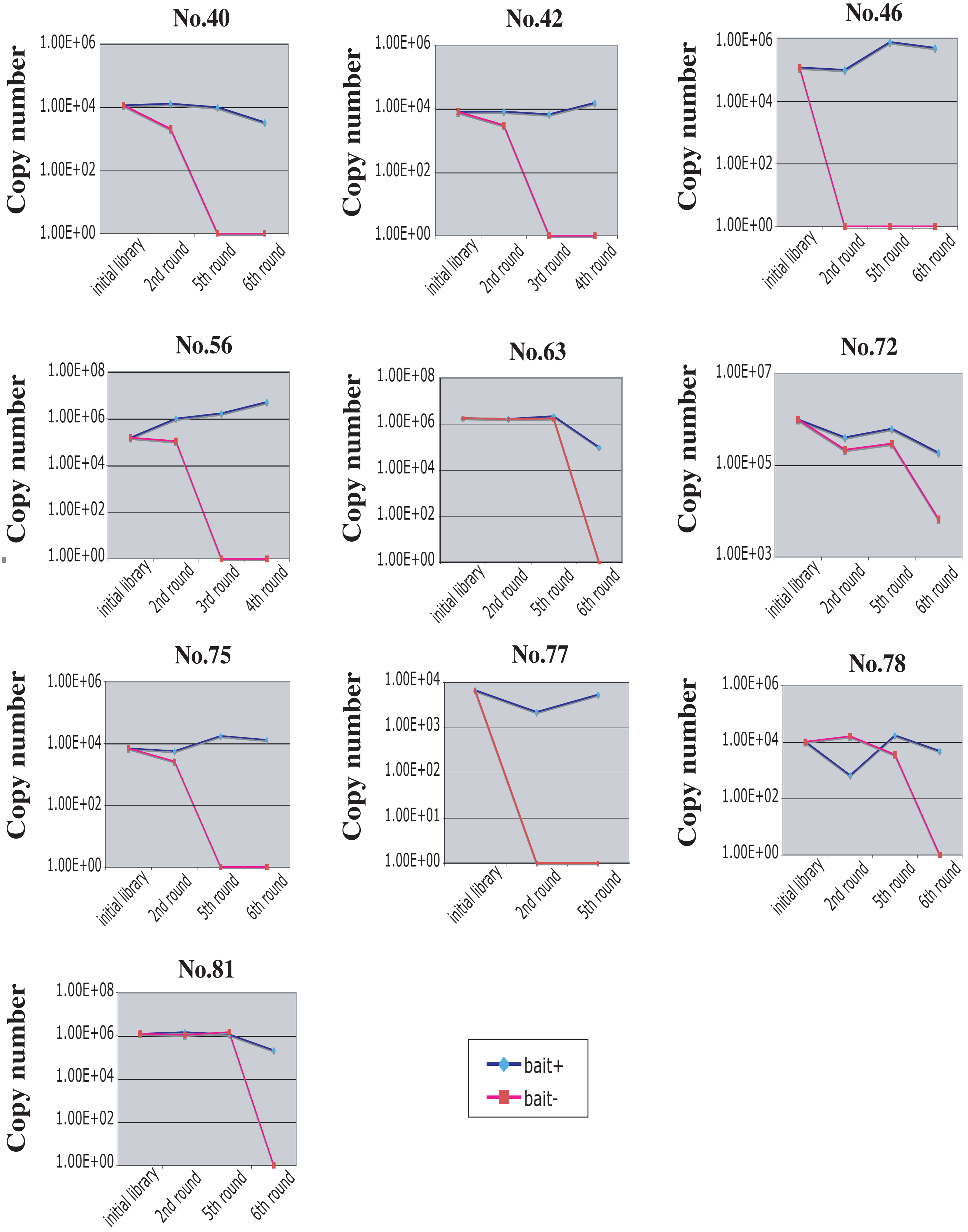
No.40
Copy number
1.00E+06
1.00E+04
1.00E+02
1.00E+00
initial library
2nd round
5th round
6th round
No.42
Copy number
1.00E+06
1.00E+04
1.00E+02
1.00E+00
initial library
2nd round
3rd round
4th round
No.46
Copy number
1.00E+06
1.00E+04
1.00E+02
1.00E+00
initial library
2nd round
5th round
6th round
No.56
Copy number
1.00E+08
1.00E+06
1.00E+04
1.00E+02
1.00E+00
initial library
2nd round
3rd round
4th round
No.63
Copy number
1.00E+08
1.00E+06
1.00E+04
1.00E+02
1.00E+00
initial library
2nd round
5th round
6th round
No.72
Copy number
1.00E+07
1.00E+05
1.00E+03
initial library
2nd round
5th round
6th round
No.75
Copy number
1.00E+06
1.00E+04
1.00E+02
1.00E+00
initial library
2nd round
5th round
6th round
No.77
Copy number
1.00E+04
1.00E+03
1.00E+02
1.00E+01
1.00E+00
initial library
2nd round
5th round
No.78
Copy number
1.00E+06
1.00E+04
1.00E+02
1.00E+00
initial library
2nd round
5th round
6th round
No.81
Copy number
1.00E+08
1.00E+06
1.00E+04
1.00E+02
1.00E+00
initial library
2nd round
5th round
6th round
bait+
bait-
