# Supplementary material for: A Comprehensive Resource of Interacting Protein Regions for Refining Human Transcription Factor Networks
Source: PLoS One. 2010 Feb 24;5(2):e9289. doi: 10.1371/journal.pone.0009289 (PMC2827538; doi:10.1371/journal.pone.0009289)
Supplement: Figure S6 — Alignment of ISTs with Pfam domains. IST-mapped regions that overlapped with any Pfam domain/motif region were aligned with the corresponding full-length proteins. ISTs and Pfam domain/motif regions in the full-length proteins are represented by solid black and green squares, respectively. Only ISTs belonging to class 1 are illustrated in the figure. Pfam domain/motif regions were obtained from the Pfam ftp site (ftp://ftp.sanger.ac.uk/pub/databases/Pfam/current_release/swisspfam.gz). The following 31 bait/prey combinations are depicted: EEF1D/EPRS, ETS1/JUN, FOS/ATF2, FOS/CABP1, FOS/JUN, FOS/JUND, FOS/HSPA1A, JUN/ATF2, JUN/CREB3, JUN/FOS, JUN/HSPA8, JUN/MAPRE3, MAX/FTH1, MAX/FUS, MAX/RPL34, MAX/RPL35, MAX/TUBA3, MDM2/APP, MDM2/CLU, MDM2/JUN, MDM2/JUND, MDM2/PKM2, MYC/KIDINS220, PAX8/ANXA7, PHB/COX6C, SCHIP1/TMSB4X, SP1/NAP1L1, SP1/TPI1, SMAD2/JUN, TAF9/FEZ1, TAF9/RPS24. (0.83 MB PDF) [file pone.0009289.s007.pdf]

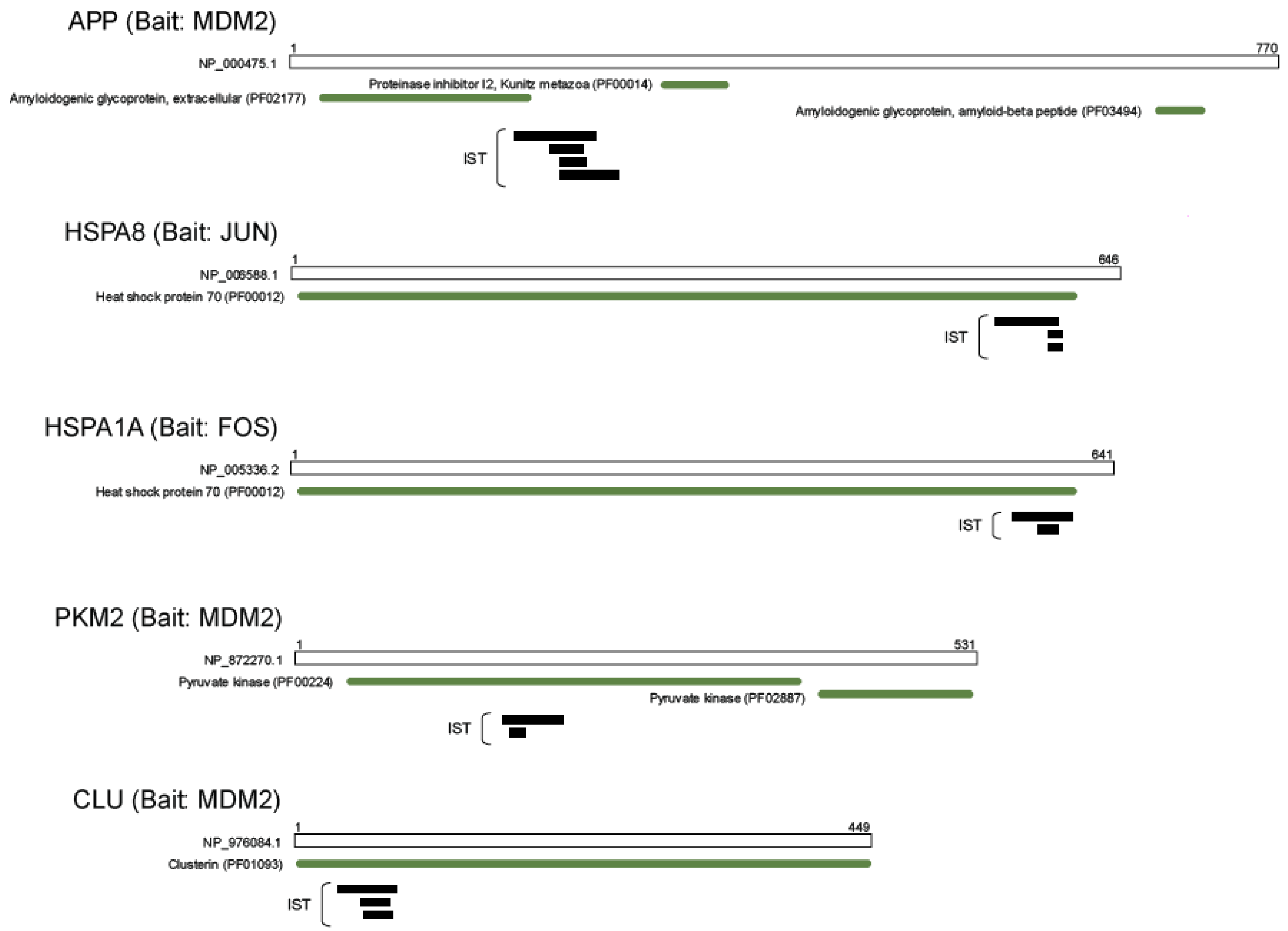

APP (Bait: MDM2)
NP_000475.1
1
770
Proteinase inhibitor I2, Kunitz metazoa (PF00014)
Amyloidogenic glycoprotein, extracellular (PF02177)
Amyloidogenic glycoprotein, amyloid-beta peptide (PF03494)
IST
HSPA8 (Bait: JUN)
NP_006588.1
1
646
Heat shock protein 70 (PF00012)
IST
HSPA1A (Bait: FOS)
NP_005336.2
1
641
Heat shock protein 70 (PF00012)
IST
PKM2 (Bait: MDM2)
NP_872270.1
1
531
Pyruvate kinase (PF00224)
Pyruvate kinase (PF02887)
IST
CLU (Bait: MDM2)
NP_976084.1
1
449
Clusterin (PF01093)
IST

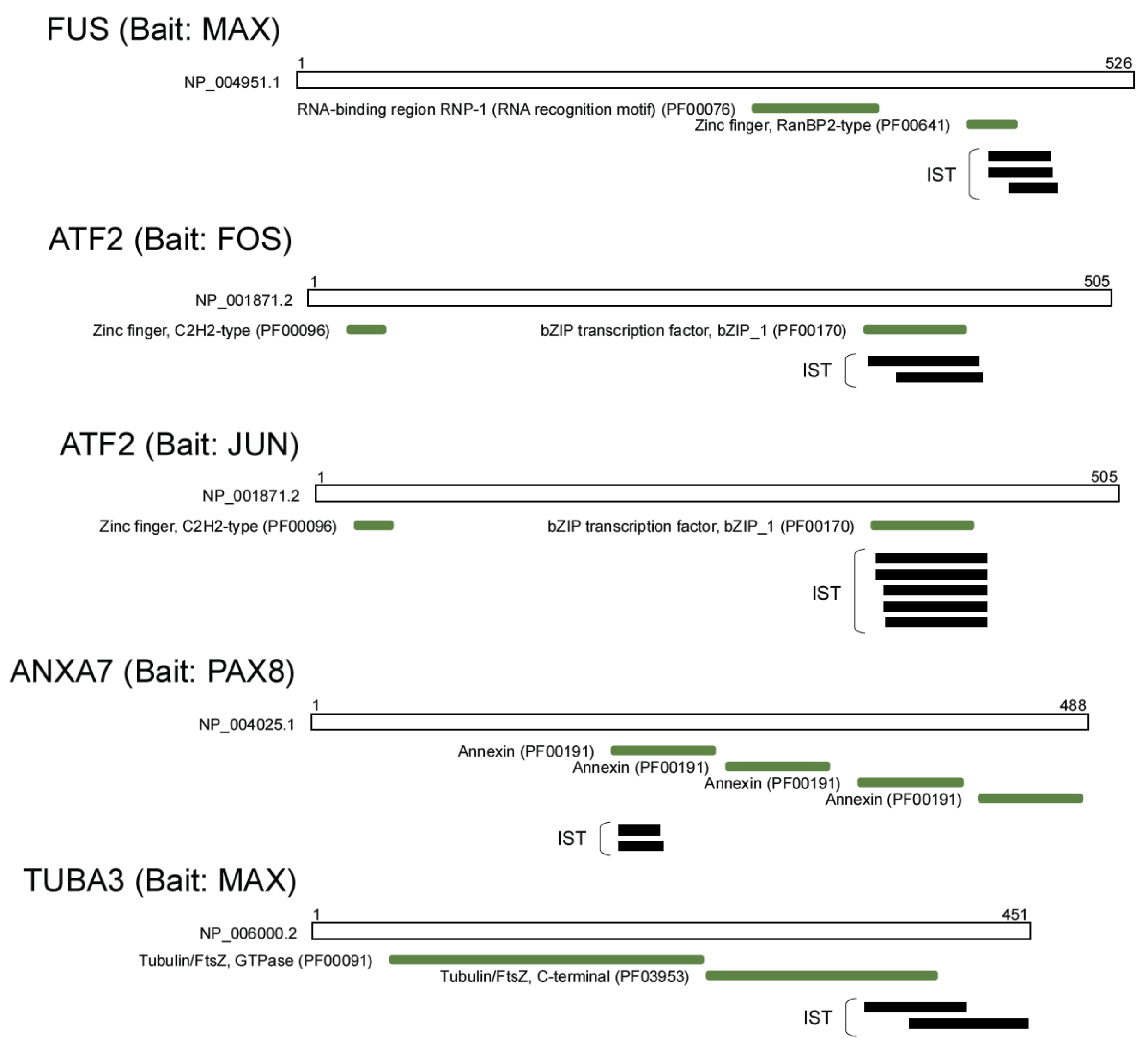
FUS (Bait: MAX)
NP_004951.1
1
526
RNA-binding region RNP-1 (RNA recognition motif) (PF00076)
Zinc finger, RanBP2-type (PF00641)
IST
ATF2 (Bait: FOS)
NP_001871.2
1
505
Zinc finger, C2H2-type (PF00096)
bZIP transcription factor, bZIP_1 (PF00170)
IST
ATF2 (Bait: JUN)
NP_001871.2
1
505
Zinc finger, C2H2-type (PF00096)
bZIP transcription factor, bZIP_1 (PF00170)
IST
ANXA7 (Bait: PAX8)
NP_004025.1
1
488
Annexin (PF00191)
Annexin (PF00191)
Annexin (PF00191)
Annexin (PF00191)
IST
TUBA3 (Bait: MAX)
NP_006000.2
1
451
Tubulin/FtsZ, GTPase (PF00091)
Tubulin/FtsZ, C-terminal (PF03953)
IST

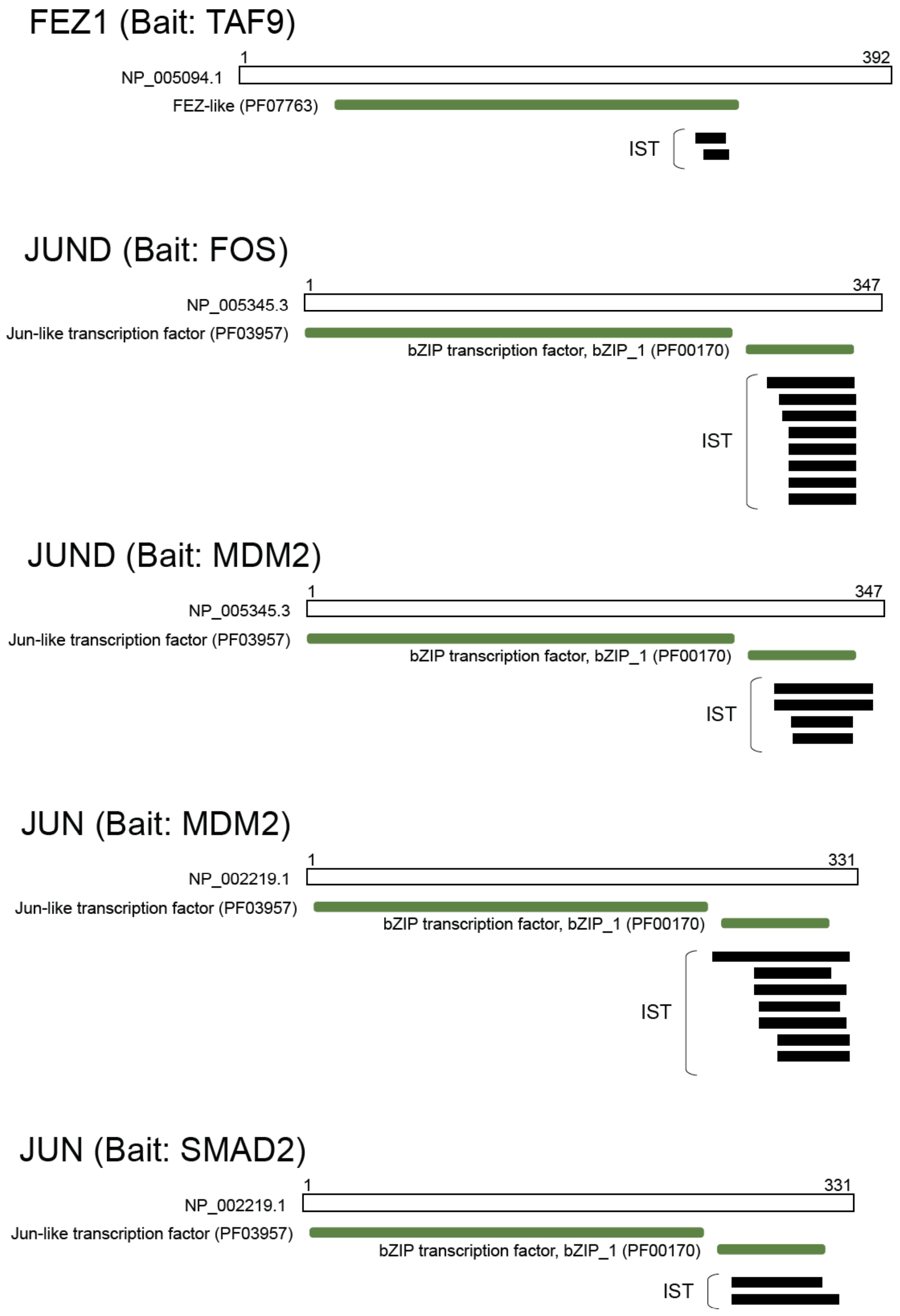

FEZ1 (Bait: TAF9)
1
392
NP_005094.1
FEZ-like (PF07763)
IST
JUND (Bait: FOS)
1
347
NP_005345.3
Jun-like transcription factor (PF03957)
bZIP transcription factor, bZIP_1 (PF00170)
IST
JUND (Bait: MDM2)
1
347
NP_005345.3
Jun-like transcription factor (PF03957)
bZIP transcription factor, bZIP_1 (PF00170)
IST
JUN (Bait: MDM2)
1
331
NP_002219.1
Jun-like transcription factor (PF03957)
bZIP transcription factor, bZIP_1 (PF00170)
IST
JUN (Bait: SMAD2)
1
331
NP_002219.1
Jun-like transcription factor (PF03957)
bZIP transcription factor, bZIP_1 (PF00170)
IST

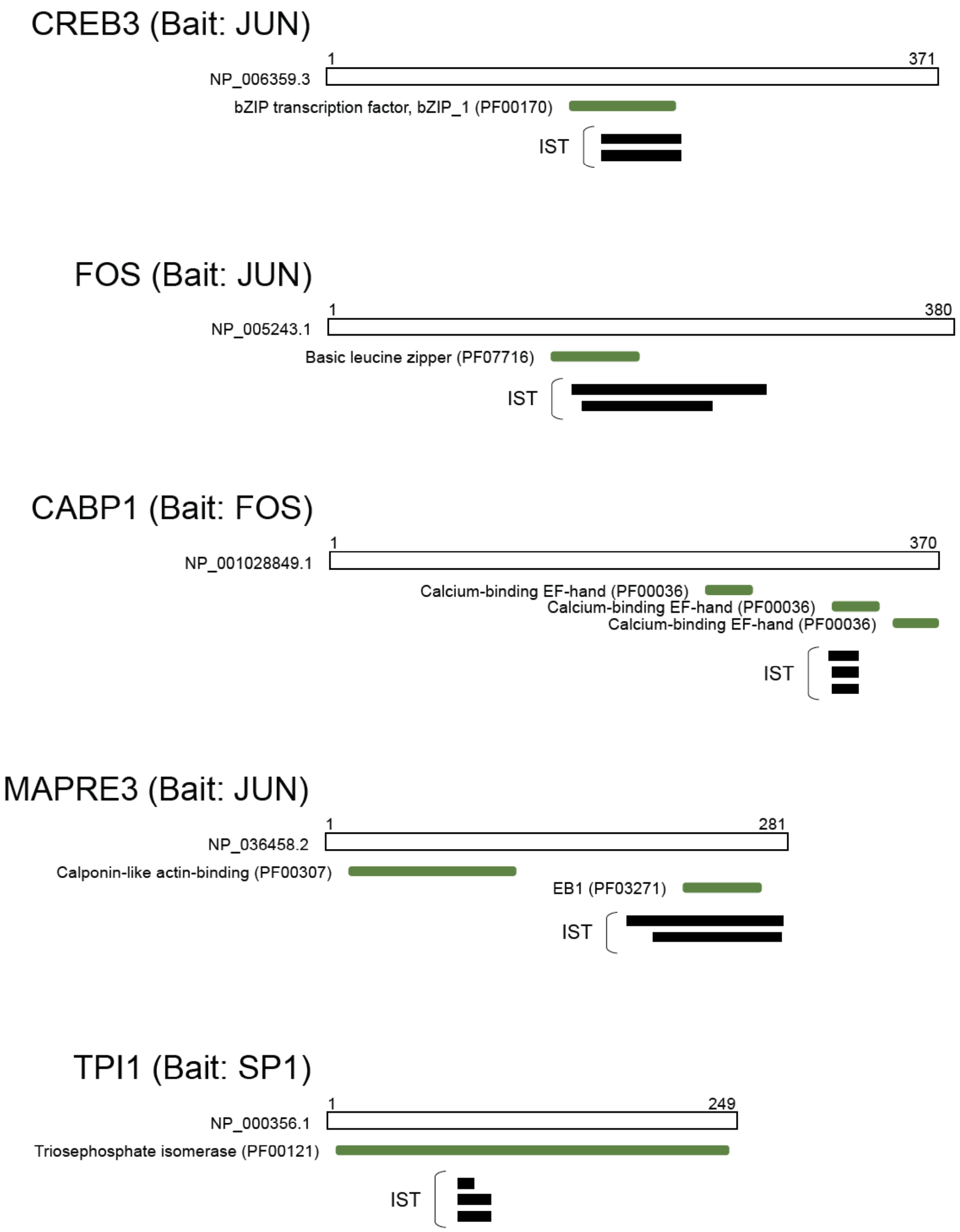

CREB3 (Bait: JUN)
1
371
NP_006359.3
bZIP transcription factor, bZIP_1 (PF00170)
IST
FOS (Bait: JUN)
1
380
NP_005243.1
Basic leucine zipper (PF07716)
IST
CABP1 (Bait: FOS)
1
370
NP_001028849.1
Calcium-binding EF-hand (PF00036)
Calcium-binding EF-hand (PF00036)
Calcium-binding EF-hand (PF00036)
IST
MAPRE3 (Bait: JUN)
1
281
NP_036458.2
Calponin-like actin-binding (PF00307)
EB1 (PF03271)
IST
TPI1 (Bait: SP1)
1
249
NP_000356.1
Triosephosphate isomerase (PF00121)
IST

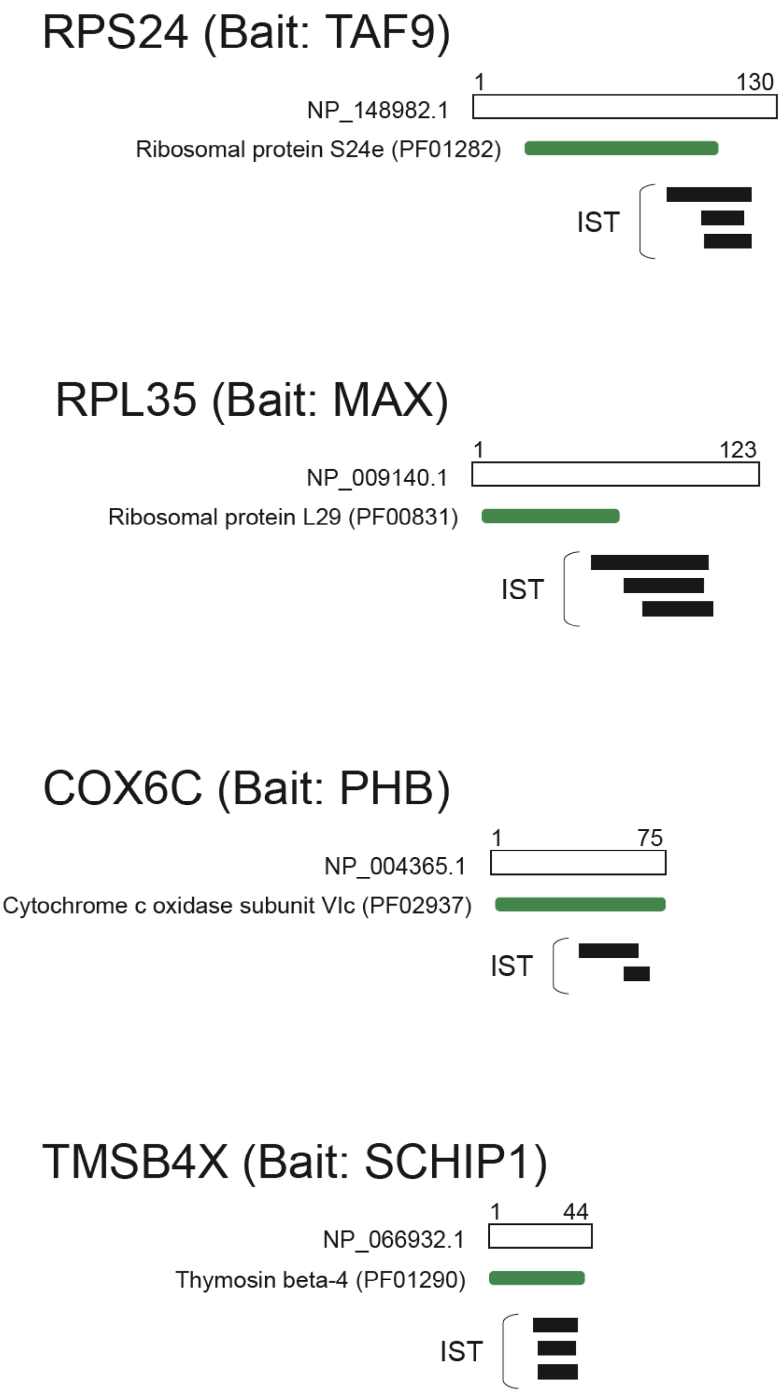
RPS24 (Bait: TAF9)
1
130
NP_148982.1
Ribosomal protein S24e (PF01282)
IST
RPL35 (Bait: MAX)
1
123
NP_009140.1
Ribosomal protein L29 (PF00831)
IST
COX6C (Bait: PHB)
1
75
NP_004365.1
Cytochrome c oxidase subunit VIc (PF02937)
IST
TMSB4X (Bait: SCHIP1)
1
44
NP_066932.1
Thymosin beta-4 (PF01290)
IST

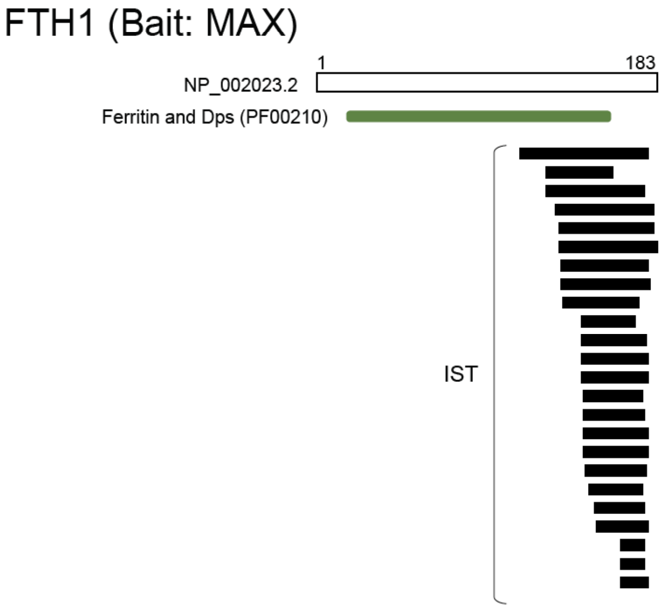
FTH1 (Bait: MAX)
1
183
NP_002023.2
Ferritin and Dps (PF00210)
IST

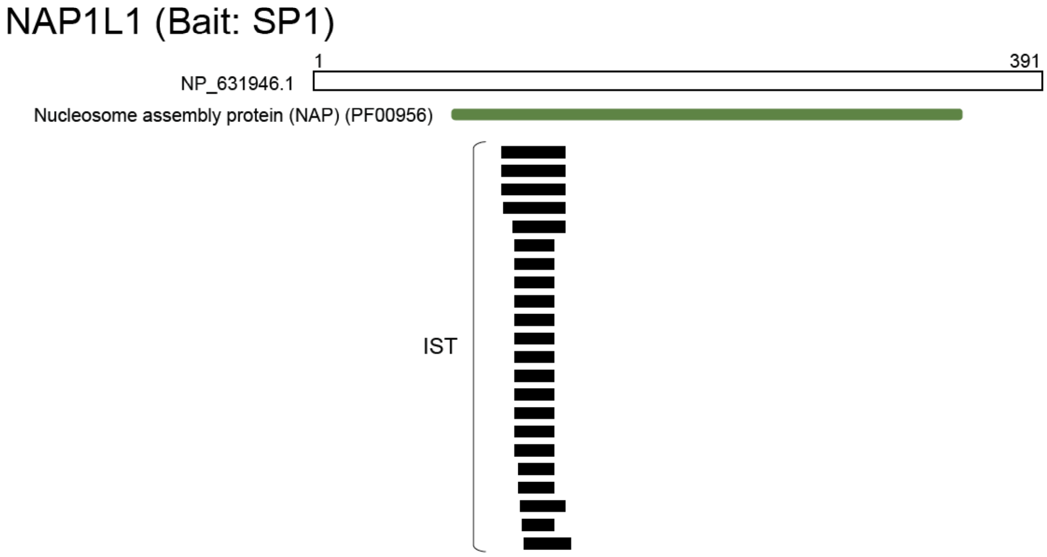
NAP1L1 (Bait: SP1)
1
391
NP_631946.1
Nucleosome assembly protein (NAP) (PF00956)
IST

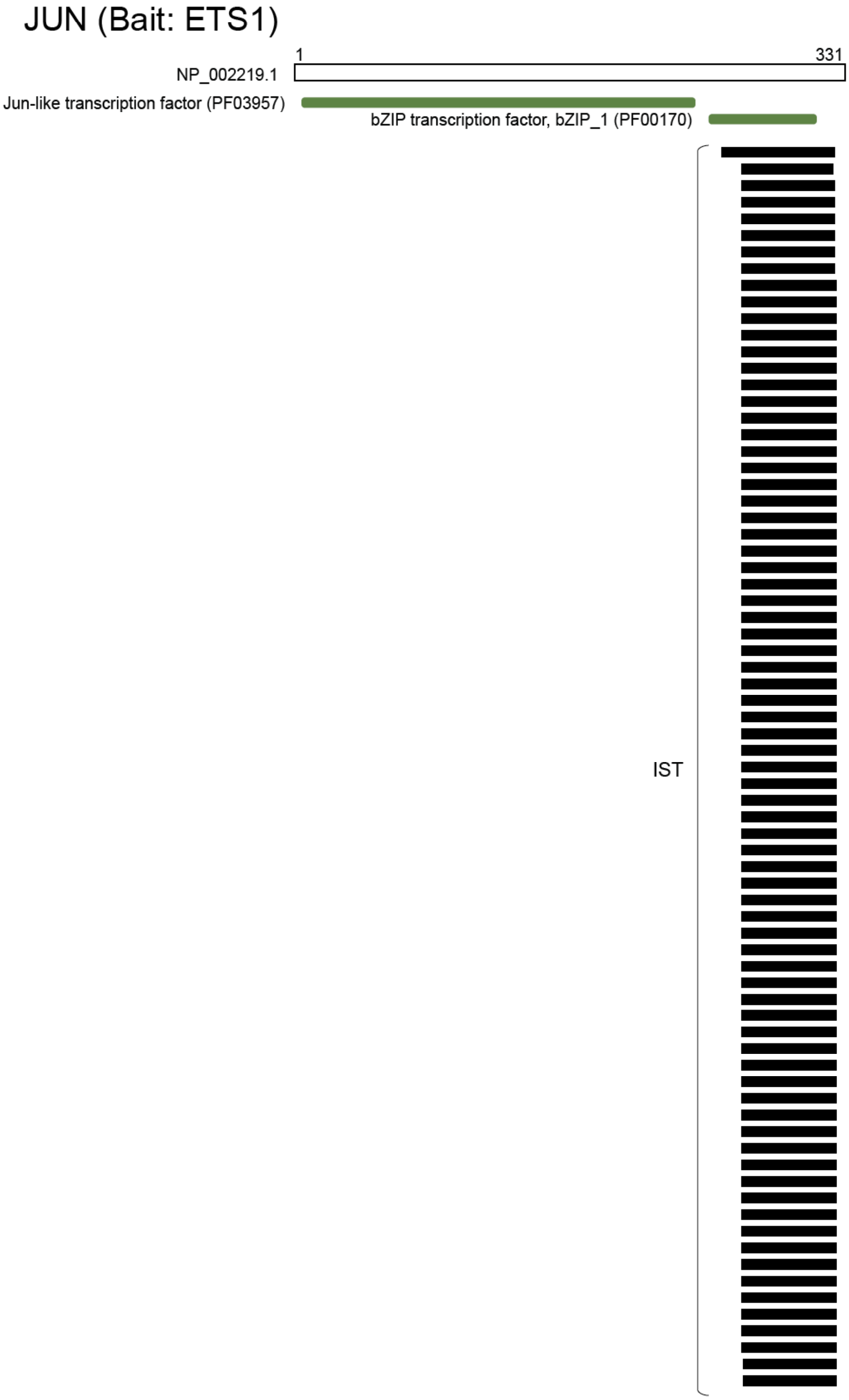

JUN (Bait: ETS1)
1
331
NP_002219.1
Jun-like transcription factor (PF03957)
bZIP transcription factor, bZIP_1 (PF00170)
IST

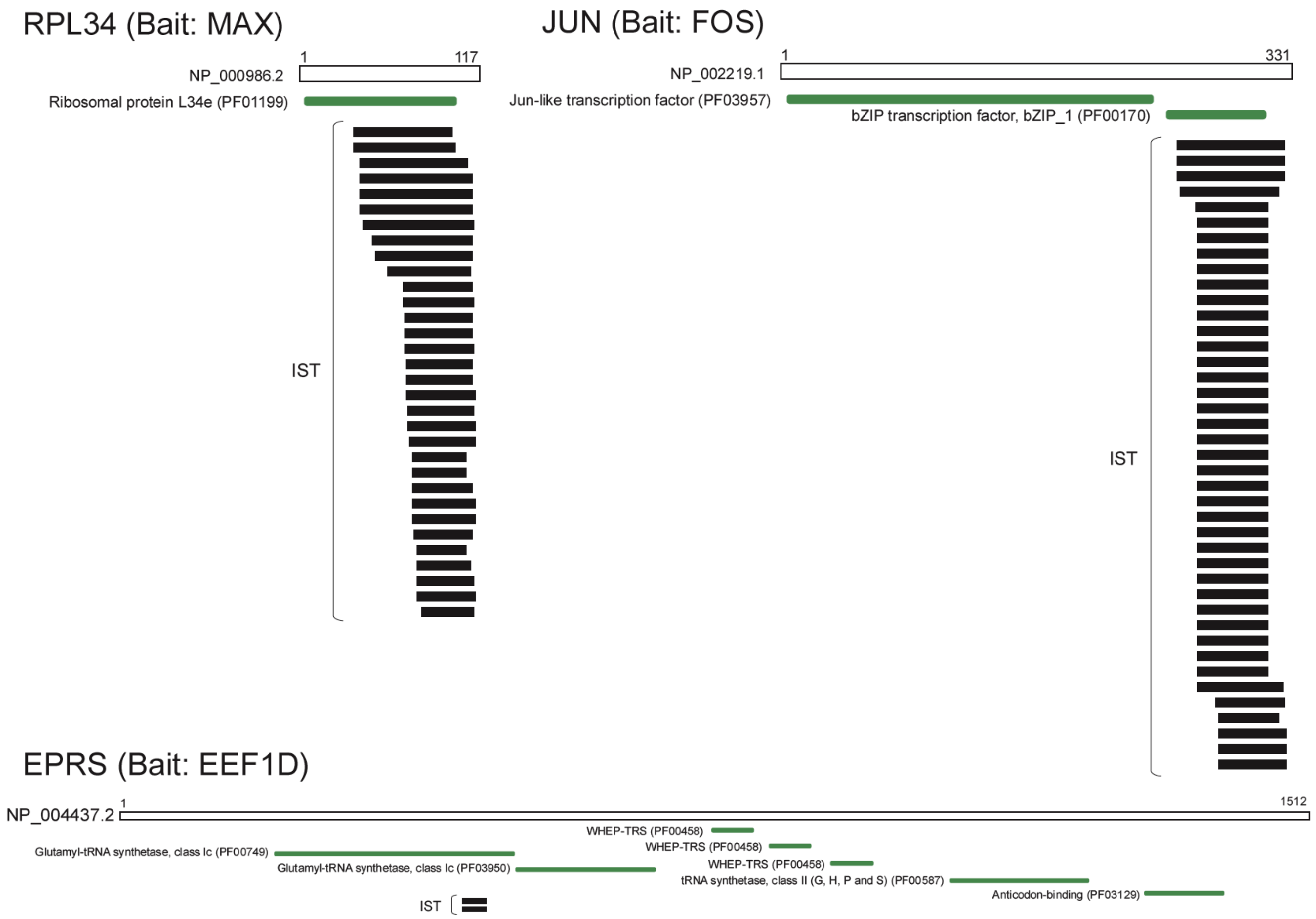

RPL34 (Bait: MAX)
1
117
NP_000986.2
Ribosomal protein L34e (PF01199)
IST
JUN (Bait: FOS)
1
331
NP_002219.1
Jun-like transcription factor (PF03957)
bZIP transcription factor, bZIP_1 (PF00170)
IST
EPRS (Bait: EEF1D)
NP_004437.2
1
1512
WHEP-TRS (PF00458)
WHEP-TRS (PF00458)
WHEP-TRS (PF00458)
Glutamyl-tRNA synthetase, class Ic (PF00749)
Glutamyl-tRNA synthetase, class Ic (PF03950)
tRNA synthetase, class II (G, H, P and S) (PF00587)
Anticodon-binding (PF03129)
IST

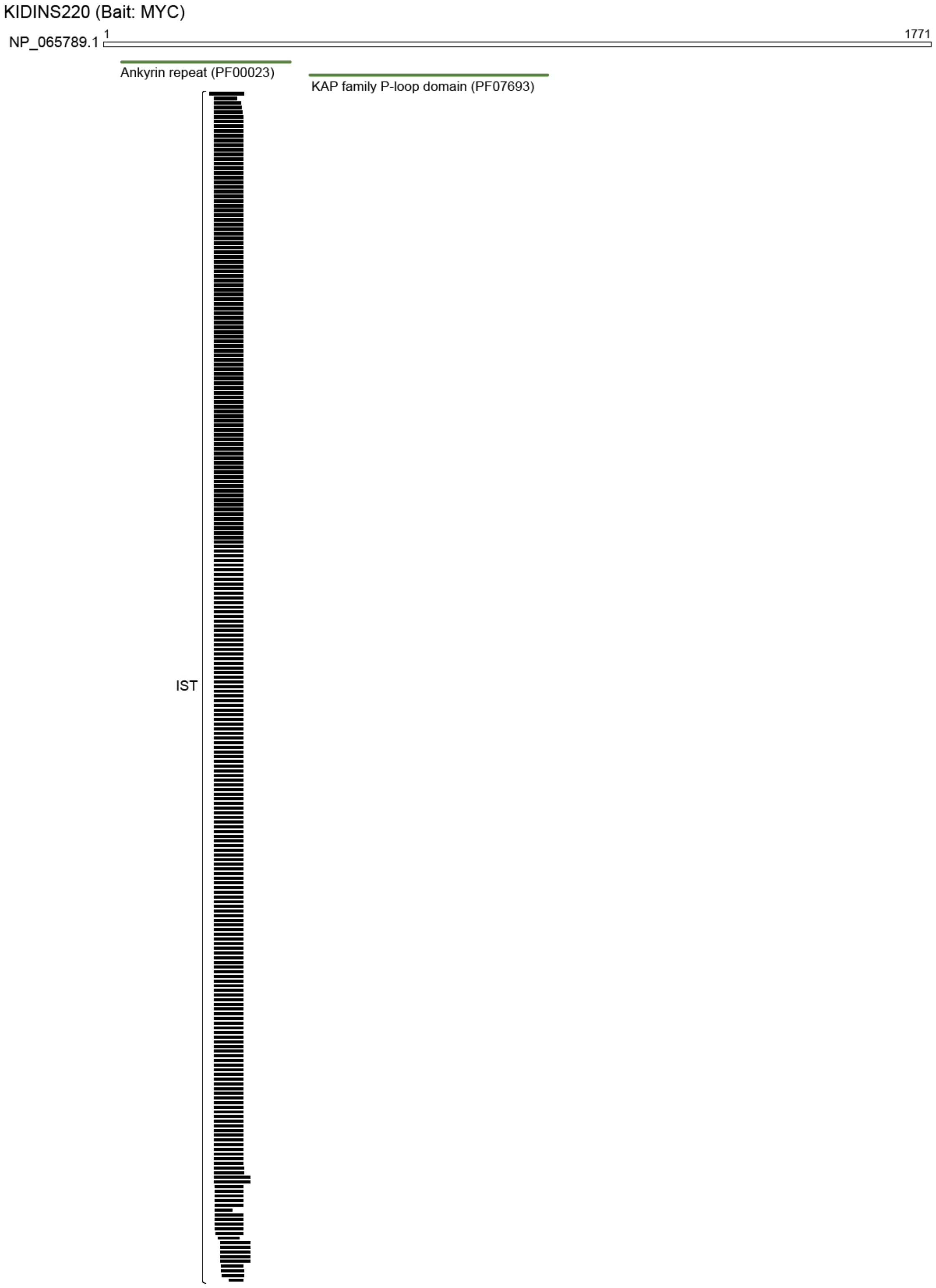

KIDINS220 (Bait: MYC)
NP_065789.1
1
1771
Ankyrin repeat (PF00023)
KAP family P-loop domain (PF07693)
IST
