# Supplementary material for: A Comprehensive Resource of Interacting Protein Regions for Refining Human Transcription Factor Networks
Source: PLoS One. 2010 Feb 24;5(2):e9289. doi: 10.1371/journal.pone.0009289 (PMC2827538; doi:10.1371/journal.pone.0009289)
Supplement: Table S2 — List of primers used in the preparation of bait protein cDNAs (see also Figure S1 and Tables S1 and S3). (0.05 MB PDF) [file pone.0009289.s015.pdf]

Table S2 List of primers used in the preparation of bait protein cDNAs

Primer name	sequence
3'ATF2_1ST_048A	CCATCTTCTCTTGAGCTCGAGACTTCCTGAGGGCTGTGAC
3'ATF7_1ST_018H	CCATCTTCTCTTGAGCTCGAGTCTGCCCGCAGACTGGGA
3'ATF7_1ST_033A_pr	CCATCTTCTCTTGAGCTCGAGTGGGCAGTCTTTATGAGCTAACAG
3'bait_hdm2_npirmer	CCATCTTCTCTTGAGCTCGAGACTATCAGATTTGTGGCGTTTTCTTTG
3'CBFB_1ST_036H	CCATCTTCTCTTGAGCTCGAGGGGTCTTGTTGTCTTCTTGCC
3'CBP_FOS_ALL	CCATCTTCTCTTGAGCTCGAGCAGGGCCAGCAGCGTGG
3'CBP_JUN_ALL	CCATCTTCTCTTGAGCTCGAGAAATGTTTGCAACTGCTGCGTTAGC
3'CBP_TP53_ALL	CCATCTTCTCTTGAGCTCGAGGTCTGAGTCAGGCCCTTC
3'COBRA1_1ST_036G_68	GGCTATGAAATTCTTTTTCCATCTTCTCTTGAGCTCGAGGAGCGGGGCAGGGGCGGGCA
3'CTNNB1_1st	CCATCTTCTCTTGAGCTCGAGAGCTTCCTTTTTAGAAAGCTGATGG
3'CTNNB1_1ST_011G	CCATCTTCTCTTGAGCTCGAGCAGGTCAGTATCAAACCAGGC
3'DEDD_1ST_008D	CCATCTTCTCTTGAGCTCGAGGGGCAATGCTTGCAGCATC
3'E2F1_1ST_039A_68	GGCTATGAAATTCTTTTTCCATCTTCTCTTGAGCTCGAGGAAATCCAGGGGGGTGAGGTCCCC
3'E2F1_1ST_039A_68	GGCTATGAAATTCTTTTTCCATCTTCTCTTGAGCTCGAGGAAATCCAGGGGGGTGAGGTCCCC
3'EEF1D_1ST_018E_68	GGCTATGAAATTCTTTTTCCATCTTCTCTTGAGCTCGAGGATCTTGTTGAAAGCTGCGATATCGACACTCTG
3'EEF1D_1ST_018E_68	GGCTATGAAATTCTTTTTCCATCTTCTCTTGAGCTCGAGGATCTTGTTGAAAGCTGCGATATCGACACTCTG
3'EEF1D_1ST_027C_pr	CCATCTTCTCTTGAGCTCGAGACTGCCAAACAGGTCAATGTCATC
3'EEF1D_1ST_027C_pr	CCATCTTCTCTTGAGCTCGAGACTGCCAAACAGGTCAATGTCATC
3'ETS1_1st	CCATCTTCTCTTGAGCTCGAGCAGGTCACACACAAAGCGG
3'ETS1_1ST_005G	CCATCTTCTCTTGAGCTCGAGCTCGTCGGCATCTGGCTT
3'FosCBPzz	GGATCTCCATTCGCCATTCA
3'FosCBPzz_68	GGATCTCCATTCGCCATTCAGGCTGC
3'FosCBPzz+1	GGATCTCCATTCGCCATTCAG
3'FosCBPzz+3	GGATCTCCATTCGCCATTCAGGC
3'GTF2F2_1ST_005B	CCATCTTCTCTTGAGCTCGAGGTCACTCTTTTCTTCTCCTTGATAGTG
3'HES4_1ST_030B	CCATCTTCTCTTGAGCTCGAGGCGCAGCCACGGCCTC
3'JUNB_1ST_005E	CCATCTTCTCTTGAGCTCGAGGAAGGCGTGTCCCTTGACCC
3'JUNB_1ST_027H_68pr	GGCTATGAAATTCTTTTTCCATCTTCTCTTGAGCTCGAGGGTCATGACCTTCTGTTTGAGCTGGGC
3'JUND_1ST_027B_68pr	GGCTATGAAATTCTTTTTCCATCTTCTCTTGAGCTCGAGGCTGAGGACTTTCTGCTTGAGCTGCG
3'LASS2_1ST_051C	CCATCTTCTCTTGAGCTCGAGGTCATTCTTACGATGGTTGTTATTGAG
3'MAX_1ST_005D	CCATCTTCTCTTGAGCTCGAGGCTGGCCTCCATCCGGAG

3'MDM2_1ST_024F	CCATCTTCTCTTGAGCTCGAGGGGGAAATAAGTTAGCACAATCATTTG
3'MTERF_1ST_054B	CCATCTTCTCTTGAGCTCGAGGGCAAATCTGCTTAACTTTTTCAATTTAG
3'MYB_1ST_030D	CCATCTTCTCTTGAGCTCGAGCATGACCAGCGTCCGGGC
3'MYB_1ST_039D_m	CCATCTTCTCTTGAGCTCGAGGACCTTCCGACGCATTGTAGAA
3'MYC_1ST_005C	CCATCTTCTCTTGAGCTCGAGCGCACAAGAGTTCCGTAGCTG
3'MYC_M1&2_1st	CCATCTTCTCTTGAGCTCGAGCGCACAAGAGTTCCGTAGC
3'MYC_M1&2_1st	CCATCTTCTCTTGAGCTCGAGCGCACAAGAGTTCCGTAGC
3'NDN_1ST_015A	CCATCTTCTCTTGAGCTCGAGGTCCTCAGAGACACTGCTGC
3'NFKB1_1st	CCATCTTCTCTTGAGCTCGAGACTTCCAGTGCCCCCTCC
3'NFKB1_1ST_024E	CCATCTTCTCTTGAGCTCGAGAATTTTGCCTTCTAGAGGTCCTTC
3'NR2F6_1ST_024C_68	GGCTATGAAATTCTTTTTCCATCTTCTCTTGAGCTCGAGCTGGCCCGAGCCGTAGGGCCA
3'NR3C1_1st	CCATCTTCTCTTGAGCTCGAGATCTCCATTATCCTTAATTTTGGGTTTAG
3'NR3C1_1ST_011H	CCATCTTCTCTTGAGCTCGAGCTTTTGATGAAACAGAAGTTTTTTGATATTTCC
3'NR4A1_1st	CCATCTTCTCTTGAGCTCGAGCACAGCACAGCGGCCTTC
3'NR4A1_1ST_015C	CCATCTTCTCTTGAGCTCGAGGAAGGGCAGCGTGTCCAT
3'NR4A2_1ST_054C	CCATCTTCTCTTGAGCTCGAGTATTCTGTATGCTAATCGAAGGACAAA
3'OPTN_1ST_051G_m	CCATCTTCTCTTGAGCTCGAGTCCTTCAGCCATCCTAATTTCAAC
3'PAX8_1ST_015H	CCATCTTCTCTTGAGCTCGAGCAGATGGTCAAAGGCCGTG
3'PCBD_1ST_024A	CCATCTTCTCTTGAGCTCGAGTGTCATGGACACTGCTACTTGTTC
3'PCBD_1ST_024A	CCATCTTCTCTTGAGCTCGAGTGTCATGGACACTGCTACTTGTTC
3'PHB_1ST_015D	CCATCTTCTCTTGAGCTCGAGCTGGGGCAGCTGGAGGA
3'PITX2_1ST_048H	CCATCTTCTCTTGAGCTCGAGCACGGGCCGGTCCAC
3'PPARG_1ST_015B	CCATCTTCTCTTGAGCTCGAGGTACAAGTCCTTGTAGATCTCCTG
3'PRKAR1A_1ST_015E68	GGCTATGAAATTCTTTTTCCATCTTCTCTTGAGCTCGAGGACAGACAGTGACACAAAACTGTTGTACTGCTG
3'RAC3_1ST_054E	CCATCTTCTCTTGAGCTCGAGGAAGACGGTGCACTTCTTCC
3'RB1_1ST_030A	CCATCTTCTCTTGAGCTCGAGTTTCTCTTCCTTGTTTGAGGTATCC
3'RB1_1ST_030A	CCATCTTCTCTTGAGCTCGAGTTTCTCTTCCTTGTTTGAGGTATCC
3'RXRB_1st_68	CCATCTTCTCTTGAGCTCGAGCTCCCTCTTCATGCCAGTGGCC
3'RXRG_1ST_021G	CCATCTTCTCTTGAGCTCGAGGGTGATCTGCAGCGGGG
3'SCHIP1_1st	CCATCTTCTCTTGAGCTCGAGCTTTGCAGGCATTTTCTCTG
3'SCHIP1_1st	CCATCTTCTCTTGAGCTCGAGCTTTGCAGGCATTTTCTCTG
3'SCHIP1_1ST_018G	CCATCTTCTCTTGAGCTCGAGCTTTGCAGGCATTTTCTCTGCCA
3'SCHIP1_1ST_018G	CCATCTTCTCTTGAGCTCGAGCTTTGCAGGCATTTTCTCTGCCA
3'SCHIP1_1ST_027A_pr	CCATCTTCTCTTGAGCTCGAGCATGTGCTTCTGCTGACTTTCAG

3'SCHIP1_1ST_027A_pr	CCATCTTCTCTTGAGCTCGAGCATGTGCTTCTGCTGACTTTCAG
3'SMAD2_1ST_030E	CCATCTTCTCTTGAGCTCGAGTGACATGCTTGAGCAACGCAC
3'SMAD2_1ST_030E	CCATCTTCTCTTGAGCTCGAGTGACATGCTTGAGCAACGCAC
3'SOD2_1ST_011E	CCATCTTCTCTTGAGCTCGAGCTTTTTGCAAGCCATGTATCTTTCAG
3'SP1_1ST_036F	CCATCTTCTCTTGAGCTCGAGGAAGCCATTGCCACTGATATTAATG
3'SP1_1ST_039G_m	CCATCTTCTCTTGAGCTCGAGAATGGACTGCAGATCTGCCACC
3'SYT_2	CTCGAGCTCAAGAGAAGATGGCTGCTGGTAATTTCCATACTGTCCCTGGTCATAT
3'TAF9_1ST_011B	GAGCTCGAGCAGATTATCATAGTCATCATCATCATCATCGTCATCATCATCATCTTCACGTTTTCTTTTCAATGCATTTGATG
3'TCF1_1ST_036B	CCATCTTCTCTTGAGCTCGAGCTGGGAGGAAGAGGCCA
3'TCF1_1ST_039E_m	CCATCTTCTCTTGAGCTCGAGGGGCAGCGCAGGTCC
3'TFCP2_1ST_048C	CCATCTTCTCTTGAGCTCGAGCTTCAGTATGATATGATAGCTATCATTG
3'TNF_1ST_030G	CCATCTTCTCTTGAGCTCGAGCAGGGCAATGATCCCAAAGTAG
3'TWIST1_1ST_030C	CCATCTTCTCTTGAGCTCGAGGTGGGACGCGGACATGGA
3'ZNF24_1ST_054F	CCATCTTCTCTTGAGCTCGAGAACTTCCACAACATTCAGAAGTTTTTCT
5'SYT/SSX_1	ATGGCTAGCATGACTGGTGGACAGCAAATGGGTCGCGGATCCATGTCTGTGGCTTTCGCGGC
5'ATF2_1ST_048A	ATGGCTAGCATGACTGGTGGACAGCAAATGGGTCGCGGATCCATGAAATTCAAGTTACATGTGAATTCTGC
5'ATF7_1ST_018H	ATGGCTAGCATGACTGGTGGACAGCAAATGGGTCGCGGATCCATGGGAGACGACAGACCGTTT
5'ATF7_1ST_033A_pr	ATGGCTAGCATGACTGGTGGACAGCAAATGGGTCGCGGATCCGCTGCAGCCTCCCGC
5'CBFB_1ST_036H	ATGGCTAGCATGACTGGTGGACAGCAAATGGGTCGCGGATCCATGCCGCGCGTCGTGC
5'COBRA1_1ST_036G_68	ATGGCTAGCATGACTGGTGGACAGCAAATGGGTCGCGGATCCATGCCGTCCCTGCAGCCCGTGG
5'CTNNB1_1ST_011G	ATGGCTAGCATGACTGGTGGACAGCAAATGGGTCGCGGATCCATGGCTACTCAAGCTGATTTGATGG
5'CTNNB1_1ST_011G	ATGGCTAGCATGACTGGTGGACAGCAAATGGGTCGCGGATCCATGGCTACTCAAGCTGATTTGATGG
5'DEDD_1ST_008D	ATGGCTAGCATGACTGGTGGACAGCAAATGGGTCGCGGATCCATGGCGGGCCTAAAGCGG
5'E2F1_1ST_039B_68m	ATGGCTAGCATGACTGGTGGACAGCAAATGGGTCGCGGATCCGCCGACTCGCTCCTGGAGCATGTG
5'EEF1D_1ST_018E_68	ATGGCTAGCATGACTGGTGGACAGCAAATGGGTCGCGGATCCATGGCTACAAACTTCCTAGCACATGAGAAGATCTG
5'EEF1D_1ST_018E_68	ATGGCTAGCATGACTGGTGGACAGCAAATGGGTCGCGGATCCATGGCTACAAACTTCCTAGCACATGAGAAGATCTG
5'EEF1D_1ST_027C_pr	ATGGCTAGCATGACTGGTGGACAGCAAATGGGTCGCGGATCCGCCACGGCCCCACAGA
5'EEF1D_1ST_027C_pr	ATGGCTAGCATGACTGGTGGACAGCAAATGGGTCGCGGATCCGCCACGGCCCCACAGA
5'ETS1_1st	ATGGCTAGCATGACTGGTGGACAGCAAATGGGTCGCGGATCCCCAATCCAGCTATGGCAGTTTC
5'ETS1_1ST_005G	ATGGCTAGCATGACTGGTGGACAGCAAATGGGTCGCGGATCCATGAAGGCGGCCGTCGAT
5'GTF2F2_1ST_005B	ATGGCTAGCATGACTGGTGGACAGCAAATGGGTCGCGGATCCATGGCCGAGCGCGGGGAA
5'HES4_1ST_030B	ATGGCTAGCATGACTGGTGGACAGCAAATGGGTCGCGGATCCATGGCCGCAGACACGCCG
5'JUNB_1ST_005E	ATGGCTAGCATGACTGGTGGACAGCAAATGGGTCGCGGATCCATGTGCACTAAAATGGAACAGCCC
5'JUNB_1ST_027H_68pr	ATGGCTAGCATGACTGGTGGACAGCAAATGGGTCGCGGATCCGCGGCCACCAAGTGCCGGAAG

5'JUND_1ST_027B_68pr	ATGGCTAGCATGACTGGTGGACAGCAAATGGGTCGCGGATCCCGCATCGCCGCCTCCAAGTGCC
5'LASS2_1ST_051C	ATGGCTAGCATGACTGGTGGACAGCAAATGGGTCGCGGATCCATGGCCGTCATTGTGGATAAACC
5'MAX_1ST_005D	ATGGCTAGCATGACTGGTGGACAGCAAATGGGTCGCGGATCCATGAGCGATAACGATGACATCGAG
5'MDM2_1ST_024F	ATGGCTAGCATGACTGGTGGACAGCAAATGGGTCGCGGATCCATGTGCAATACCAACATGTCTGTACC
5'MTERF_1ST_054B	ATGGCTAGCATGACTGGTGGACAGCAAATGGGTCGCGGATCCATGCAGAGCCTTTCCTTAGGAC
5'MYB_1ST_030D	ATGGCTAGCATGACTGGTGGACAGCAAATGGGTCGCGGATCCATGGCCCGAAGACCCCG
5'MYB_1ST_039D_m	ATGGCTAGCATGACTGGTGGACAGCAAATGGGTCGCGGATCCCTCATCAAGGGTCCTTGGACCAAA
5'MYC_1ST_005C	ATGGCTAGCATGACTGGTGGACAGCAAATGGGTCGCGGATCCATGCCCCTCAACGTTAGCTTC
5'MYC_m2_1st	ATGGCTAGCATGACTGGTGGACAGCAAATGGGTCGCGGATCCAAGAGGCGAACACACAACGTC
5'NDN_1ST_015A	ATGGCTAGCATGACTGGTGGACAGCAAATGGGTCGCGGATCCATGTCAGAACAAAGTAAGGATCTGAG
5'NFKB1_1st	ATGGCTAGCATGACTGGTGGACAGCAAATGGGTCGCGGATCCCCTGAACAAATGTTTCATTTGGATCC
5'NFKB1_1ST_024E	ATGGCTAGCATGACTGGTGGACAGCAAATGGGTCGCGGATCCATGGCAGAAGATGATCCATATTTGGG
5'NR2F6_1ST_024C_68	ATGGCTAGCATGACTGGTGGACAGCAAATGGGTCGCGGATCCATGGCCATGGTGACCGGCGGCT
5'NR3C1_1st	ATGGCTAGCATGACTGGTGGACAGCAAATGGGTCGCGGATCCGATCTGTCCAAAGCAGTTTCACTC
5'NR3C1_1ST_011H	ATGGCTAGCATGACTGGTGGACAGCAAATGGGTCGCGGATCCATGGACTCCAAAGAATCATTAACTCC
5'NR4A1_1ST_015C	ATGGCTAGCATGACTGGTGGACAGCAAATGGGTCGCGGATCCATGCCCTGTATCCAAGCCCAA
5'NR4A1_1ST_015C	ATGGCTAGCATGACTGGTGGACAGCAAATGGGTCGCGGATCCATGCCCTGTATCCAAGCCCAA
5'NR4A2_1ST_054C	ATGGCTAGCATGACTGGTGGACAGCAAATGGGTCGCGGATCCATGCCTTGTGTTCAGGCGCA
5'OPTN_1ST_051G_m	ATGGCTAGCATGACTGGTGGACAGCAAATGGGTCGCGGATCCAAGGCAGACCTGTTGGGCA
5'O'T7_FOS_ALL	ACAATTACTATTTACAATTACAATGGCTAGCATGACTGGTGGACAGCAAATGGGTCGCGGATCCATGATGTTCTCGGGCTTCAACGC
5'O'T7_JUN_ALL	ACAATTACTATTTACAATTACAATGGCTAGCATGACTGGTGGACAGCAAATGGGTCGCGGATCCATGACTGCAAAGATGGAAACGACC
5'O'T7_TP53_ALL	ACAATTACTATTTACAATTACAATGGCTAGCATGACTGGTGGACAGCAAATGGGTCGCGGATCCATGGAGGAGCCGCAGTC
5'PAX8_1ST_015H	ATGGCTAGCATGACTGGTGGACAGCAAATGGGTCGCGGATCCATGCCTCACAACTCCATCAGA
5'PCBD_1ST_024A	ATGGCTAGCATGACTGGTGGACAGCAAATGGGTCGCGGATCCATGGCTGGCAAAGCACACAG
5'PCBD_1ST_024A	ATGGCTAGCATGACTGGTGGACAGCAAATGGGTCGCGGATCCATGGCTGGCAAAGCACACAG
5'PHB_1ST_015D	ATGGCTAGCATGACTGGTGGACAGCAAATGGGTCGCGGATCCATGGCTGCCAAAGTGTTTGAGTC
5'PITX2_1ST_048H	ATGGCTAGCATGACTGGTGGACAGCAAATGGGTCGCGGATCCATGGAGACCAACTGCCGCAA
5'PPARG_1ST_015B	ATGGCTAGCATGACTGGTGGACAGCAAATGGGTCGCGGATCCATGGTTGACACAGAGATGCCATTC
5'PRKAR1A_1ST_015E68	ATGGCTAGCATGACTGGTGGACAGCAAATGGGTCGCGGATCCATGGAGTCTGGCAGTACCGCCGC
5'RAC3_1ST_054E	ATGGCTAGCATGACTGGTGGACAGCAAATGGGTCGCGGATCCATGCAGGCCATCAAGTGCGT
5'RB1_1ST_039C_m	ATGGCTAGCATGACTGGTGGACAGCAAATGGGTCGCGGATCCATGAACACTATCCAACAATTAATGATGATTTTA
5'RXRB_1ST_021H_68	ATGGCTAGCATGACTGGTGGACAGCAAATGGGTCGCGGATCCATGTCTTGGGCCGCTCGCCCG
5'RXRB_1ST_021H_68	ATGGCTAGCATGACTGGTGGACAGCAAATGGGTCGCGGATCCATGTCTTGGGCCGCTCGCCCG
5'RXRG_1ST_021G	ATGGCTAGCATGACTGGTGGACAGCAAATGGGTCGCGGATCCATGTATGGAAATTATTCTCACTTCATGAAGTTTC

5'SCHIP1_1st	ATGGCTAGCATGACTGGTGGACAGCAAATGGGTCGCGGATCCGACAGTGGCAGTGATAAGGAC
5'SCHIP1_1st	ATGGCTAGCATGACTGGTGGACAGCAAATGGGTCGCGGATCCGACAGTGGCAGTGATAAGGAC
5'SCHIP1_1ST_018G	ATGGCTAGCATGACTGGTGGACAGCAAATGGGTCGCGGATCCATGGAGAGGTCCGGGCAG
5'SCHIP1_1ST_018G	ATGGCTAGCATGACTGGTGGACAGCAAATGGGTCGCGGATCCATGGAGAGGTCCGGGCAG
5'SCHIP1_1ST_027A_pr	ATGGCTAGCATGACTGGTGGACAGCAAATGGGTCGCGGATCCCCTCATATAAGTGAATGCTTGATGAAAAG
5'SCHIP1_1ST_027A_pr	ATGGCTAGCATGACTGGTGGACAGCAAATGGGTCGCGGATCCCCTCATATAAGTGAATGCTTGATGAAAAG
5'SMAD2_1ST_030E	ATGGCTAGCATGACTGGTGGACAGCAAATGGGTCGCGGATCCATGTCGTCCATCTTGCCATTCAC
5'SMAD2_1ST_039F_m	ATGGCTAGCATGACTGGTGGACAGCAAATGGGTCGCGGATCCCGACACACCGAGATCCTAAC
5'SOD2_1ST_011E	ATGGCTAGCATGACTGGTGGACAGCAAATGGGTCGCGGATCCATGTTGAGCCGGGCAGTGTG
5'SP1_1ST_036F	ATGGCTAGCATGACTGGTGGACAGCAAATGGGTCGCGGATCCATGAGCGACCAAGATCACTCC
5'SP1_1ST_039G_m	ATGGCTAGCATGACTGGTGGACAGCAAATGGGTCGCGGATCCAGTGAAGGAAGGGGCTCGG
5'SP6(O')T7	GAATTTAGGTGACACTATAGAAACAATTACTATTTACAATTACAATGGCTAGCATGACTGGTGGACAG
5'SP6(O')T7_68	GAATTTAGGTGACACTATAGAAACAATTACTATTTACAATTACAATGGCTAGCATGACTGGTGGACAGCAAATG
5'TAF9_1ST_005A	ATGGCTAGCATGACTGGTGGACAGCAAATGGGTCGCGGATCCATGGAGTCTGGCAAGACGGC
5'TCF1_1ST_036B	ATGGCTAGCATGACTGGTGGACAGCAAATGGGTCGCGGATCCATGGTTTCTAAACTGAGCCAGC
5'TCF1_1ST_036B	ATGGCTAGCATGACTGGTGGACAGCAAATGGGTCGCGGATCCATGGTTTCTAAACTGAGCCAGC
5'TFCP2_1ST_048C	ATGGCTAGCATGACTGGTGGACAGCAAATGGGTCGCGGATCCATGGCCTGGGCTCTGAAG
5'TNF_1ST_030G	ATGGCTAGCATGACTGGTGGACAGCAAATGGGTCGCGGATCCATGAGCACTGAAAGCATGATCCG
5'TWIST1_1ST_030C	ATGGCTAGCATGACTGGTGGACAGCAAATGGGTCGCGGATCCATGATGCAGGACGTGTCCAG
5'ZNF24_1ST_054F	ATGGCTAGCATGACTGGTGGACAGCAAATGGGTCGCGGATCCATGTCTGCACAGTCAGTGGAAG
human_5'T7+mdm2	ATGGCTAGCATGACTGGTGGACAGCAAATGGGTCGCGGATCCATGTGCAATACCAACATGTCTGTAC
5'TAF9_2ND_012B	GACTATGATAATCTGCTCGAGCTCAAGAGAAGATGG
5'baitCBP_primer	CTCGAGCTCAAGAGAAGATGG
