# Supplementary material for: A Comprehensive Resource of Interacting Protein Regions for Refining Human Transcription Factor Networks
Source: PLoS One. 2010 Feb 24;5(2):e9289. doi: 10.1371/journal.pone.0009289 (PMC2827538; doi:10.1371/journal.pone.0009289)
Supplement: Table S3 — List of PCR programs used for amplification of bait cDNA templates. (0.12 MB PDF) [file pone.0009289.s016.pdf]

Table S3. List of PCR programs used for amplification of bait cDNA templates.

Program number	program
1	1cycle of 98°C for 2min, 15cycles of 98°C for 30sec 62°C for 30sec and 72°C for 2min, and 1cycle of 72°C for 15min.
2	1cycle of 98°C for 2min, 15cycles of 98°C for 30sec, 62°C for 30sec and 72°C for 4min, and 1cycle of 72°C for 15min.
3	1cycle of 98°C for 1min, 4cycles of 98°C for 30sec and 72°C for 1min, 4cycles of 98°C for 30sec and 70°C for 1min, and 24cycles of 98°C for 30sec and 68°C for 1min
4	1cycle of 98°C for 1min, 4cycles of 98°C for 30sec and 72°C for 2min, 4cycles of 98°C for 30sec and 70°C for 2min, and 24cycles of 98°C for 30sec and 68°C for 2min
5	1cycle of 98°C for 1min, 4cycles of 98°C for 30sec and 72°C for 3min, 4cycles of 98°C for 30sec and 70°C for 3min, and 24cycles of 98°C for 30sec and 68°C for 3min
6	1cycle of 98°C for 1min, 4cycles of 98°C for 30sec and 72°C for 2min, 4cycles of 98°C for 30sec and 70°C for 2min, and 5 cycles of 98°C for 30sec and 68°C for 2min
7	1cycle of 98°C for 2min, and 25cycles of 98°C for 15sec 62°C for 30sec and 68°C for 3min.
8	1cycle of 95°C for 1min, 35cycles of 95°C for 1min, 60°C for 1min, and 72°C for 1min

See also Figure S1 and Tables S1-2.
