# Supplementary material for: A Comprehensive Resource of Interacting Protein Regions for Refining Human Transcription Factor Networks
Source: PLoS One. 2010 Feb 24;5(2):e9289. doi: 10.1371/journal.pone.0009289 (PMC2827538; doi:10.1371/journal.pone.0009289)
Supplement: Table S8 — Increase of LC PPIs by the IVV and Y2H data sets. (0.05 MB PDF) [file pone.0009289.s021.pdf]

Table S8. Increase of LC PPIs by the IVV and Y2H data sets.

		A		*B*		Ratio of *A* to *B*	
	Baits	PPIs newly added	Nodes (genes) newly added	LC PPIs	Nodes (genes) originally presented	PPIs	Genes
IVV (Core)	50	932	612	1240	748	0.75	0.82
Y2H (TF)[§]	116	372	172	776	542	0.48	0.32

The expansion of the available PPI network with the IVV core data set and the Y2H data set is shown within each TF network. § : Interactions are limited to those that include bait proteins having the GO assignment 'transcription regulator activity' or 'transcription factor activity'.
