# Supplementary material for: A Comprehensive Resource of Interacting Protein Regions for Refining Human Transcription Factor Networks
Source: PLoS One. 2010 Feb 24;5(2):e9289. doi: 10.1371/journal.pone.0009289 (PMC2827538; doi:10.1371/journal.pone.0009289)
Supplement: Table S9 — Comparison of the network characteristics. (0.06 MB PDF) [file pone.0009289.s022.pdf]

Table S9. Comparison of the network characteristics.

	Degree distribution: $P(k) \propto k^{-r}$	Clustering coefficient: $C(k) \propto k^{-r}$	Neighbor connectivity: $N(k) \propto k^{-r}$	Mean path length	Mean clustering coefficient
IVV (Core)	r=1.21 (R^2=0.72)	r=0.82 (R^2=0.52)	r=0.85 (R^2=0.53)	4.35	0.005
Y2H (Stelzl *et al.*)	r=1.65 (R^2=0.91)	r=0.51 (R^2=0.33)	r=0.32 (R^2=0.55)	4.86	0.006
Y2H (Rual *et al.*)	r=1.54 (R^2=0.89)	r=0.81 (R^2=0.54)	r=0.38 (R^2=0.66)	4.36	0.028
LC (50 TFs)[§]	r=1.07 (R^2=0.73)	r=1.01 (R^2=0.78)	r=0.70 (R^2=0.73)	3.49	0.099

r indicates the slope of the approximate straight line on a log-log plot graph corresponding to each distribution.

§ : Indicates LC PPIs that have direct relationships with the 50 TF proteins used as bait in the IVV experiment.
