# Supplementary material for: A Comprehensive Resource of Interacting Protein Regions for Refining Human Transcription Factor Networks
Source: PLoS One. 2010 Feb 24;5(2):e9289. doi: 10.1371/journal.pone.0009289 (PMC2827538; doi:10.1371/journal.pone.0009289)
Supplement: Table S10 — Frequencies of tissue-specific PPIs in the IVV core data set. (0.04 MB PDF) [file pone.0009289.s023.pdf]

Table S10. Frequencies of tissue-specific PPIs in the IVV core data set.

Tissue	$N_{specific}$	C (50 × TissueSpecificGenes)	$N_{specific}/C$
Brain	128	45,200	2.8E-03
Liver	10	25,300	4.0E-04
Lung	7	24,800	2.8E-04
Kidney	5	14,050	3.6E-04
Heart	4	13,550	3.0E-04
